# Phenolic Profile and Antioxidant, Antibacterial, and Antiproliferative Activity of *Juglans regia* L. Male Flowers

**DOI:** 10.3390/molecules27092762

**Published:** 2022-04-26

**Authors:** Natalia Żurek, Agata Pawłowska, Karolina Pycia, Dorota Grabek-Lejko, Ireneusz Tomasz Kapusta

**Affiliations:** 1 Department Food Technology and Human Nutrition, Institute of Food Technology and Nutrition, University of Rzeszow, 4 Zelwerowicza St., 35-601 Rzeszow, Poland; nzurek@ur.edu.pl (N.Ż.); agpawlowska@ur.edu.pl (A.P.); kpycia@ur.edu.pl (K.P.); 2 Department of Bioenergetics, Food Analysis and Microbiology, Institute of Food Technology and Nutrition, University of Rzeszow, 4 Zelwerowicza St., 35-601 Rzeszow, Poland; dgrabek@ur.edu.pl

**Keywords:** *Juglans regia*, flowers, polyphenol compounds, antioxidant activity, anticancer activity, antimicrobial activity

## Abstract

*Juglans regia* L., walnut, is a large, long-living tree, cultivated in temperate climates around the world. It is highly appreciated for its nutritional kernels and high-quality timber. Its barks, leaves, and husk are used as dyes and in folk medicine as herbal remedies for several diseases. From a biological and chemical standpoint, relatively little is known about the male flowers of the tree. Therefore, the aim of the study was to evaluate the phenolic profile as well as in vitro antioxidant, antimicrobial, and antiproliferative activity of male *Juglans regia* L. flowers. Phenolic content was determined by UPLC/PDA/MS/MS analyses; antioxidant activity was assessed by five different methods; antimicrobial activity was evaluated against the six most common pathogenic strains of Gram-positive and Gram-negative bacteria, and antiproliferative properties were assessed against six cell lines. Most of the analyses carried out in this study were performed for the first time for this raw material. *J. regia* flower extract was characterized by a strong ability to scavenge DPPH˙ free radicals, hydroxyl radicals, and chelating metal ions. Among the examined bacterial strains and neoplastic lines, the strongest antimicrobial activity was shown against *S. aureus*, *L. monocytogenes*, and *B. cereus*, and cytotoxic activity against breast cancer, glioblastoma, and astrocytoma cells. Male *J. regia* flowers have also been found to be a rich source of phenolic compounds. The content of polyphenols in the extract was 4369.73 mg/100 g d.w., and 24 compounds from the group of flavonoids, phenolic acids, and juglunosides were identified. Additionally, a strong correlation between the content of polyphenols and the antioxidant capacity and cytotoxic activity was observed. This is why the tested *J. regia* flowers are an excellent source of effective natural antioxidant, antibacterial, and chemopreventive compounds that have potential to be used in the pharmaceutical or food industries.

## 1. Introduction

Plant polyphenols are a wide group of secondary metabolites that can range from simple molecules, such as phenolic acids, to highly polymerized compounds such as tannins. During the last few decades, there has been growing interest in plant phenols, especially flavonoids, due to their wide range of biological effects, including antioxidant, anti-inflammatory, antiallergic, and antibacterial properties. Indeed, the results of epidemiological studies have proven that there is a correlation between increased consumption of fruits and vegetables and reduced risk of cardiovascular diseases and certain types of cancer [1].

*Juglans regia* L., walnut (*Juglandaceae* family), is a species native to Central Asia, Europe, and North America [2]. In most walnut-growing countries, their production is focused on the nuts [3]. The kernels are consumed all over the world on their own as a snack but can also be added to salads, pastas, breakfast cereals, soups, and baked goods [4]. They are also used to make walnut oil [5]. According to the International Nut and Drug Fruit Council (INC), in 2019/2020, overall walnut production reached 965,402 tonnes (kernel basis) [6]. The composition and pro-health properties of the kernels are well described. They are abundant in fat, including polyunsaturated fatty acids, proteins, vitamins, minerals, and phytochemical components, such phenolic acids and flavonoids, with potential health effects against aging, cancers, inflammation, diabetes, and neurologic illnesses [4,7,8,9,10,11,12]. However, almost all of the parts of the tree are rich in bioactive components, e.g., the leaves have been shown to have high content of phenolic components with antibiotic and antioxidative effects [13]; the bark, branches, and exocarp of the immature green fruit have been used to treat gastric, liver, and lung cancer, as well as possessing astringent, anthelmintic, depurative, bactericide, diuretic, digestive, laxative, stimulant, detergent, and insecticidal properties [14]; the seed coat has been used for healing wounds [15]; the shell of *J. regia* has been used in Calabria folk medicine to heal malaria [16]; the flowers have been shown to possess remarkable anti-hypoxic, anti-inflammatory, antioxidant, and antidepressant activities, partially due to the high content of phenols and flavonoids [17].

Relatively little is known about the male flowers of *J. regia*. The male flowers of walnut are grouped into inflorescences (catkins), which develop laterally on a shoot, mainly in its basal part. Each catkin consists of 100–160 individual male flowers, containing 2–32 stamens each. The length of the catkin and the number of flowers per catkin depend on the walnut species and the genotype. Compared to other anemophilous species, a walnut tree produces a relatively small number of catkins (85–300 per tree) [18]. In China, the male inflorescence of walnut has been known as a longevity food [19]. In the minority ethnic areas, in the Guizhou and Yunnan Provinces, it was a traditional vegetable, used as an ingredient in fried dishes, soup, and Chinese vegetable salads [20]. In Poland, the walnut catkins are used to prepare infusions, tinctures, liquors, jams, and confectionaries for pies.

Limited chemical studies have been conducted on the male flowers of this plant. There are a few reports describing the total content of phenolics and flavonoids as well their biological activities [17,21]. However, only one study has addressed the quali–quantitative analysis of a species grown in India [22]. The most essential polyphenolic constituents so far identified in *J. regia* flowers are phenolic acids (e.g., derivatives of caffeic acids), flavonoids, and glycosides (rutin, quercetin diglucoside, quercetin 3-*O*-glucoside). Therefore, the subject of the research was to perform a complete phytochemical study of *J. regia* L. male inflorescences, including qualitative and quantitative analysis of their content by ultra-performance liquid chromatography (UPLC) coupled to photodiode array detection (PDA) and tandem mass spectrometry (MS/MS); to determine the total content of polyphenols, flavonoids, and proantocynidins as well as antioxidant activity by a battery of in vitro tests; and to evaluate their anticancer properties against six cell lines and antibacterial performance against the six most common pathogenic strains of Gram-positive and Gram-negative bacteria, in order to fill a gap in relation to the scientific knowledge about walnut male flowers and to highlight their potential as food.

## 2. Results and Discussion

### 2.1. Effects of Total Phenolic, Flavonoid, and Proanthocyanidin Contents

The obtained results of total phenolic (TP), total flavonoid (TF), and total proanthocyanidin (TPA) content in extracts of *J. regia* male flowers are presented in Table 1. The concentration of TP in the analyzed extract was 248.33 ± 2.33 mg of gallic acid equivalent per g of dry weight (mg GAE/g d.w.), while the contents of TF and TPA were 111.01 ± 1.03 mg of quercetin equivalent per g of dry weight (mg QE/g d.w.) and 16.66 ± 0.70 mg of cyanidin equivalent per 1 g of dry weight (mg CYE/g d.w.), respectively.

Thus far, no studies have been reported on the TPA content of *J. regia* flowers. On the other hand, in our research, the content of two groups of compounds—TP and TF—was several hundred times higher than in the other studies submitted. Muzaffer et al. (2018) estimated that in ethanol extracts of male *J. regia* flowers collected in India, the content of TP and TF was 1.45 ± 0.02 mg GAE/g d.w. and 1.30 ± 0.03 mg QE/g d.w., respectively. Comparable results were obtained in the earlier study by Nabawi et al. (2011) and in the recent investigations of Pop et al. (2021) [17,22,23]. TP contents were also determined in other aerial parts of the *J. regia* tree. In the extracts of various varieties of walnut leaves, it ranged from 65 mg/g to 194 mg/g GAE d.w. [13]. In turn, Pycia et al. (2019) found that the content of polyphenols in dry walnut seeds of various cultivars growing in Poland reached from 8.2 mg/g up to 20.9 mg/g GAE d.w. [12]. The reported TP value is therefore lower than that of the walnut catkin extract, which may indicate that the flowers are a rich natural source of polyphenols compared to other parts of the walnut.

### 2.2. Antioxidant Activity

*J. regia* flowers contain many types of polyphenolic compounds, which exert antioxidant effects through various mechanisms. Based on these mechanisms, a battery of in vitro tests was used to fully analyze the antioxidant capacity of *J. regia* flowers. The antioxidant activity was determined using the 2,2-diphenyl-1-picrylhydrazyl (DPPH˙) radical scavenging activity assay, copper ion reduction assay (CUPRAC), metal chelating ability (ChA), and the activity of scavenging superoxide (O^2●−^) and hydroxyl (OH^−^) radicals (Table 2, Figure S1, Supplementary Materials). These methods are considered to be an important indicator of the antioxidant potential of plant samples [24]. Based on the available literature, there are only three reports in which the antioxidant potential of *J. regia* flowers was analyzed [17,22,23]. In this study, the free radical scavenging DPPH˙ showed the lowest IC_50_ value, which corresponds to the highest antioxidant activity. The calculated half maximum inhibitory concentration (IC_50_) value was determined as 22.34 ± 2.70 µg/mL and it was higher compared to ascorbic acid, used as a positive control (5.00 ± 0.01 µg/mL). Equally, a low IC_50_ value indicating high antioxidant activity was demonstrated for the chelating capacity test (71.69 ± 0.02 µg/mL), the superoxide scavenging (147.06 ± 0.27 μg/mL) test, and the hydroxyl radicals test (41.85 ± 0.09 µg/mL). However, for the last method, based on the reduction of copper ions using the CUPRAC method, the antioxidant activity of the extract, expressed in Trolox equivalents, was 3.33 ± 0.01 mmol TE/g.

The obtained IC_50_ value for DPPH˙ radical scavenging activity was around 3 and 100 times lower compared to the values obtained in other previously developed studies assessing the antioxidant activity of flowers [17,22,23]. Compared to the antioxidant activity of leaves, nuts, and walnut shells assessed with the DPPH test, the obtained results are also around 10 times lower [8,13]. However, there are no scientific reports on the CUPRAC method, chelating capacity, superoxide scavenging, and hydroxyl radical activity of *J. regia* flower extracts. This paper is the first one to assess the antioxidant potential of flowers using these methods. When referring to the results obtained for other morphological parts of the walnut, in the shell, the ability to scavenge superoxide and hydroxyl radicals was two times higher than in our study [25]; in the leaves, instead, the outcomes were two times lower for the superoxide scavenging, and almost 25 times higher for the activity of hydroxyl radicals [26].

Thus, the obtained results indicate that *J. regia* flowers have a high ability to scavenge free radicals, which may be due to the high content of bioactive compounds. Moreover, the outcomes showed a strong correlation between analyzed phenolic compounds and their antioxidant activities. Significant correlations (*p* = 0.05) were found between TP and the DPPH method (r > 0.997), and additionally among TPA and the hydroxyl radical scavenging assay (r > 0.999).

### 2.3. Antibacterial Potential

The antibacterial activity of *J. regia* flower extract was investigated against Gram-positive and Gram-negative bacteria. As can be seen in Table 3, the extract possessed antibacterial properties against all tested bacterial strains. The minimum inhibitory concentration (MIC) ranged from 0.3125 to 20 mg/mL. The highest antibacterial potential could be observed for Gram-positive bacteria. Among them, the most sensitive was *S. aureus* (MIC 0.3125 mg/mL). Gram-negative bacteria were less susceptible. The most resistant strain was *K. pneumoniae* with MIC 20 mg/mL. In most cases, the minimum bactericidal concentration (MBC) was the same as the MIC concentration, with one exception only. The MBC value (2.5 mg/mL) for *L. monocytogenes* was higher than the MIC value (0.625 mg/mL).

No statistical correlation was found between the antimicrobial activity and the chemical profile.

Thus far, the antimicrobial activity of walnut flower extracts has been evaluated in only one study by Muzaffer et al. (2018) [22]. In this study, the MIC concentrations against Gram-positive *S. aureus* and Gram-negative *E. coli* were approximately 14 and 3 times higher, respectively, than in our study. No reports have been submitted for the remaining strains of bacteria. The results of the antibacterial activity of *J. regia* extract obtained in this study are comparable with the results of Pereira et al. (2007) and Zakavi et al. (2013), respectively, for the leaves and bark of the walnut tree [8,27].

### 2.4. Viability of Cancer Cell Lines

**Figure 1:**
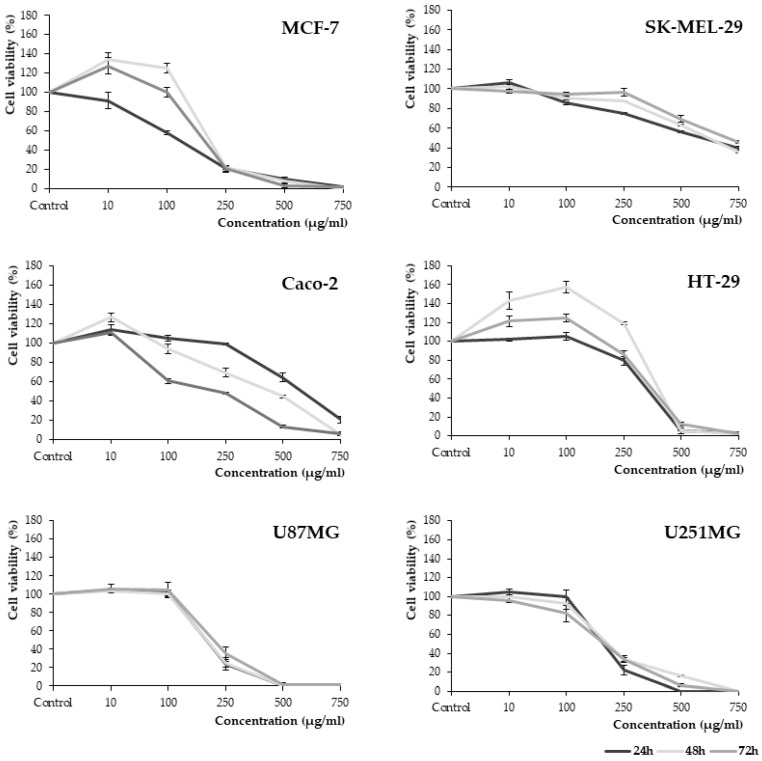
Cytotoxic effect of different concentrations (10–750 µg/mL) of *J. regia* flower extracts on breast adenocarcinoma (MCF-7), melanoma (SK-Mel-29), colorectal adenocarcinoma (Caco-2; HT-29), glioblastoma (U87MG), and astrocytoma cell lines (U251MG). All cells were treated for 24, 48, and 72 h. The number of viable control (non-treated) cells at each time point served as 100%. Graphs represent mean values ± SD from three independent experiments.

The cytotoxic effects of *J. regia* flower extract were investigated on six human cancer cell lines. The cells were subjected to various concentrations of flower extract for 24, 48, and 72 h and effects analyzed by means of the 3-(4,5-dimethylthiazol-2-yl)-5-(3-carboxymethoxyphenyl)-2-(4-sulfophenyl)-2H-tetrazolium (MTS) assay. The flower extract showed a dose-dependent inhibitory effect on the growth of all cell lines (see Figure 1). As shown in Table 4, the extract inhibited most of the viability of MCF-7, U87MG, and U251MG cells. The lowest recorded IC_50_ values were 132.35 ± 8.73 µg/mL, 195.79 ± 8.57 µg/mL, and 200.42 ± 4.98 µg/mL for the MCF-7, U251MG, and U87MG cell lines, respectively. The lowest sensitivity to the tested extract was noted after the treatment of melanoma cells (SK-MEL-29). IC_50_ values ranged from 593.62 ± 16.88 to 706.21 ± 11.01 µg/mL for incubation times from 24 to 72 h. This is the first report of the antiproliferative activity of walnut flower extracts.

Analyzing the anticancer activity of other parts of the walnut tree, Carvalho et al. (2010), studying the effect of green husk and leaf extract on the viability of human renal epithelial cancer cells, A-498, obtained results comparable to the effects of flowers on the astrocytoma line. However, for the Caco-2 line, the results were comparable to the results obtained in this study [28]. Nevertheless, Yang et al. (2009), treating Caco-2 cells with nut extracts, obtained values almost three times higher than in this study. Equally, low sensitivity to seeds was demonstrated in relation to the hepatocellular carcinoma (HepG2) cell line [29]. These results demonstrate the high antitumor activity of the walnut flower extract. The inhibition of cancer cell proliferation by plant extracts largely depends on the total amount of phenolic compounds, individual phenolic classes, or the corresponding phenolic compound. The above-mentioned studies also show the relationship between the content of polyphenols and the anticancer effect. Our results show a strong Pearson correlation between TP and MCF-7 and U87MG cancer cell viability—r > 0.926 and 0.945—while U251MG cell viability was strongly dependent on TF and the correlation was r > 0.925.

### 2.5. Identification and Quantification of Phenolic Compounds

The extract of *J. regia* male flowers was analyzed by means of ultra-performance liquid chromatography (UPLC) coupled to photodiode array detection (PDA) and tandem quadrupole mass spectrometry (TQD) with electrospray ionization (ESI). The extract was considered from both qualitative and quantitative points of view. The components were identified on the basis of literature data and by comparison of their retention times (Rt), elution orders, spectrometric data, and photodiode array with reference compounds. All of the chromatographic, spectroscopic, and spectrometric data, together with the quantities of single compounds, are presented in Table 5.

The analyses revealed the presence of 24 compounds in the extract. Nine represented the group of flavonoids, 12 compounds belonged to phenolic acid derivatives, and three were recognized as juglanoside isomers. Flavonoids could be distinguished from the other compounds by their characteristic ultraviolet–visible (UV–Vis) absorption spectra. The methanol spectra of flavones and flavonols exhibit two major absorption peaks in the region 240–400 nm [30]. The other phenols identified in the flower extract were phenolic acids and their esters, mainly caffeoyl derivatives. Caffeoyl derivatives could be distinguished by the characteristic UV spectra with two peak maximum absorption bands at 200sh and 325–330 nm and a typical fragmentation pattern with a daughter ion at *m/z* 179 [31]. Three compounds were determined as coumarin derivatives. Two of them were isomers of coumaryl-quinic acid with different substituent positions and the latter one was an ester of coumaryl-quinic acid with caffeic acid. All of the derivatives gave the same fragmentation pattern, with the predominant ion at *m/z* 163. In turn, juglanoside isomers were already identified in other parts of the *J. regia* species [32]. The most abundant constituents were quercetin 3-*O*-glucoside, followed by quercetin diglucoside and 5-*O*-caffeoylquinic acid (Table 5).

Chrzanowski et al. (2011), in male inflorescences of *J. regia* L., identified caffeic, ferulic, chlorogenic, and vanillic acids. Interestingly, vanillic acid was the predominant compound (359.5 μg/g d.w.), and it was not observed in our study. This difference in the composition might depend on the species, growing conditions, such as soil, geographical and environmental conditions during development, degree of maturity at harvest, and genetic differences [33].

However, all the identified classes of compounds were already recognized in different parts of *J. regia* L. Zhang et al. (2020), in female flowers of walnut, reported the presence of 29 phenolic compounds [32]. Ellagic acid and ferulic acid isomers were the main components in the group of phenolic acids, and the flavonoids were represented mainly by afzelin, myricitrin, and quercitrin. In the leaves of walnuts of various varieties growing in Portugal, Pereira et al. (2007) identified several quinic acid derivatives and quercetine glucosides. Quercetin 3-galactoside and 3-caffeoylquinic acid dominated the polyphenol spectrum in the analyzed extracts. Moreover, Stampar et al. (2006) identified 13 polyphenols in the green husk of unripe walnuts, the most important of which were chlorogenic acid, caffeic acid, ferulic acid, synapic acid, syringic acid, vanillic acid, catechin, and epicatechin [34]. Hama et al. (2016) determined phenolic compounds in the defatted kernel, green husk, and leaves of walnut. Nine phenolic compounds with 1–4, naphthoquinone, and juglone were detected [35]. Persic et al. (2018), in the pellicle walnut kernels of various varieties, identified sixteen phenolic compounds: six derivatives of hydroxybenzoic acids, five derivatives of hydroxycinnamic acids, and five flavonoids. Seven derivatives/isomers of dicarboxylic acids, mainly isomers/derivatives of glansreginin A, were also detected in the polyphenol profiles. However, vanillic acid hexoside was the dominant component [36]. On the other hand, Zhang et al. (2020) found ellagic and quinic acid derivatives as main constituents of walnut pellicles [32]. In dry walnut kernels, instead, Colaric et al. (2005) identified several polyphenols, such as chlorogenic acid, caffeic acid, *p*-coumaric acid, ferulic acid, sinapic acid (hydroxycinnamic acids), ellagic acid, syringic acid (hydroxybenzoic acids) as well as syring-aldehyde (hydroxybenzoic aldehyde) and juglone [37]. Similarly, Pycia et al. (2019a) recognized 18 derivatives of hydroxybenzoic and hydroxycinnamic acids in the dry seeds of walnuts of various varieties grown in Poland [38].

## 3. Materials and Methods

### 3.1. Materials and Reagents

Quercetin (≥95%), gallic acid (≥98%), cyanidin chloride (≥98%), LiChroprep RP-18 (40–63 µm), neocuproine (≥98%), ferrozine (≥97%), NBT (nitrotetrazolium blue chloride), PMS (phenazine methosulfate, ≥90%), NADH (*β*-Nicotinamide adenine dinucleotide, reduced disodium salt hydrate, ≥97%), 2-Deoxy-d-ribose, EDTA (ethylenediaminetetraacetic acid disodium salt dihydrate), Dulbecco’s Modified Eagle Medium, McCoy’s 5A Medium, fetal bovine serum, antibiotics (100 U/mL penicillin, 100 U/mL streptomycin), 0.25% trypsin–EDTA (1×), Dulbecco’s Phosphate Buffered Saline, and Mueller–Hinton Broth were purchased from Sigma-Aldrich (Darmstadt, Germany). The AQueous CellTiter 96 Non-Radioactive Cell Proliferation Test was purchased from Promega (Madison, WI, USA). Reference standard compounds for UPLC analyses were obtained from Extrasynthese (Lyon, France) and Sigma-Aldrich (Darmstadt, Germany). All other chemicals were purchased from Chempur (Piekary Śląskie, Poland).

### 3.2. Plant Material

The male *Juglans regia* L. flowers were collected from trees in the Subcarpathian region in Poland in May 2018. The material was lyophilized (ALPHA 1–2 LD plus Martin Christ Gefriertrocknungsanlagen GmbH, Germany), ground, and stored at −20 °C prior to analysis.

### 3.3. Preparation of Extract

The extract of *J. regia* flowers was prepared according to our previous reports [39]. In brief, the powdered material was mixed in a 1:10 ratio with methanol (50%, *v*/*v*) and left for 24 h. The mixture was centrifuged, the solution was decanted, and the residue was extracted again with methanol (80%, *v*/*v)* using ultrasound (Sonic 10 ultrasonic bath, Polsonic, Poland) at 30 °C for 30 min. The solution was centrifuged, and the supernatants were combined and concentrated by means of a rotary evaporator (R-215 Rotavapor System, Buchi, Switzerland) at 40 °C. The concentrated extracts were subjected to fractionation on an adsorber resin LiChroprep RP-18 (40–63 µm) column, using water and methanol as eluents. The resulting methanol extract was evaporated in a vacuum, freeze-dried, and stored at −20 °C until analysis.

### 3.4. Determination of Total Phenolic, Flavonoid, and Total Proanthocyanidin Content

The total phenolic (TP) content was evaluated using the method described by Gao et al. (2000) [40]. Two mL of distilled water and 0.2 mL of Folin–Ciocalteau reagent were added to the plant extracts. After 5 min, the mixture was combined with 1.0 mL sodium carbonate (20%, *w*/*v*) and left for 1 h. Then, the absorbance was measured at a wavelength of 765 nm using a UV–Vis spectrometer (Type UV2900, Hitachi, Japan).

The total flavonoid (TF) content was estimated by following the procedure developed by Chang et al. (2020) [41]. The plant extracts were mixed with 1.5 mL ethanol (96%), 0.1 mL aluminum chloride (10%, *w*/*v*), 0.1 mL sodium acetate (1 M), and 2.8 mL distilled water. After 30 min, the absorbance was measured at 415 nm.

The total proanthocyanidin (TPA) content was determined according to the method described by Porter et al. (1985) [42]. The solutions of the test extracts were combined with 3 mL *n*-BuOH (in 35% HCl, 95:5, *v*/*v*) and 0.1 mL of iron (III) ammonium sulfate (2%, in 2 M HCl). The samples were incubated at 95 °C for 45 min and then cooled to 25 °C and the absorbance was measured at 550 nm.

The results of TP, TF, and TPA contents were expressed as milligram equivalent of gallic acid per gram of dry weight (mg GAE/g d.w.), milligram equivalent of quercetin per gram of dry weight (mg QE/g d.w.), and milligram equivalent of cyanidin chloride per gram of dry weight (mg CYE/g d.w.), respectively.

### 3.5. Determination of Antioxidant Activity

#### 3.5.1. DPPH˙ Radical Scavenging Activity

The scavenging activity of flower extracts on DPPH˙ radicals was determined according to the method of Blois (1958) and Desmarchelier et al. (1997) [43,44]. Briefly, 2.4 mL of a methanolic DPPH solution (0.1 mM) was mixed with plant extracts and left for 30 min. After this time, the absorbance was measured at 517 nm, a curve % inhibition versus concentration was plotted, and the IC_50_ value was calculated. Ascorbic acid was used as a positive control.

#### 3.5.2. Chelating Ability of Ferrous Ions

The ability of the extracts to chelate iron ions (Fe^2+^) was assessed according to the method described by Mosmann (1983), with some modifications [45]. In detail, various concentrations of the extracts were added to 0.2 mL of iron II sulfate (0.1 mM) and the reaction was started by adding 0.4 mL ferrozine solution (0.25 mM). After incubation for 10 min at room temperature, the absorbance was measured at 562 nm. The inhibition of ferrozine–Fe^2+^ complex formation was calculated as the IC_50_. EDTA was used as a positive control.

#### 3.5.3. Superoxide (O^2●−^) Radical Scavenging Activity Assay

Superoxide radical scavenging activity was measured based on the method described by Robak and Gryglewski (1988) [46]. In this experiment, different concentrations of extracts were mixed with 1.0 mL NBT (150 µM in 0.1 M phosphate buffer, pH 7.4) and 1.0 mL NADH (468 µM) and the reaction was initiated by adding 1.0 mL PMS (60 µM). After 5 min, the absorbance was measured at a wavelength of 560 nm. The ability of the test extract to inhibit the production of a superoxide radical was expressed as the IC_50_. Ascorbic acid was used as a positive control.

#### 3.5.4. Hydroxyl (OH˙) Radical Scavenging Activity Assay

Hydroxyl radical scavenging activity was evaluated by the method of Halliwell et al. (1987) [47]. Briefly, 0.9 mL mixture of 2-deoxyribose (0.2 mM in 0.2 M phosphate buffer, pH 7.4), iron ammonium sulfate (1.0 mM), EDTA (1.04 mM), ascorbic acid (1.0 mM), and perhydrol (0.1 M) were added to the plant extracts. The solution was kept for 1 h at 37 °C, and then 1.0 mL trichloroacetic acid (2.8%, *w*/*v*) and 0.5 mL thiobarbituric acid (1%, *w*/*v*) were added and samples were heated to 100 °C for 15 min. After cooling to room temperature, the absorbance was measured at 532 nm. The ability of the test extracts to inhibit hydroxyl radical production was expressed as IC_50_. Quercetin was used as a positive control.

#### 3.5.5. Determination of Copper Ion Reduction by CUPRAC Method

The CUPRAC test was determined by a spectrophotometric method described by Apak et al. (2006) [48]. In a test tube, 1.0 mL neocuproine solution (7.5 mM), 1.0 mL copper chloride (10 mM), and acetate buffer (1 M, pH 7.0) were mixed, and then the plant extracts were added. The resulting mixture was stored for 30 min, and after this time, the absorbance was measured at 450 nm. The results were expressed as Trolox equivalent per g of dry weight (mmol TE/g d.w.). Ascorbic acid was used as a positive control.

### 3.6. Determination of Minimum Inhibitory Concentration (MIC) and Minimum Bactericidal Concentration (MBC)

The antibacterial potential of the *J. regia* extract was analyzed against six bacterial strains causing food poisoning diseases: three Gram-positive—*Staphylococcus aureus* ATCC 25923, *Listeria monocytogenes* CCM 4699, and *Bacillus cereus* CCM2010—and three Gram-negative ones—*Klebsiella pneumoniae* ATCC 700600, *Salmonella enterica*, and *Escherichia coli* ATCC 25922 according to [49]. Briefly, bacterial strains were subcultured overnight at 37 °C in Mueller–Hinton agar, and then suspended in physiological saline solution to a cell density of OD600 nm—0.132—which corresponds to 0.5 McFarland turbidity standard. For the MIC determination, bacterial solutions were diluted 150× in double-concentrated Mueller–Hinton broth to the final concentration of 1–5 × 10^6^ CFU per mL. A series of twofold dilutions were prepared from *J. regia* flower extract and mixed with an equal volume (100 µL) of previously prepared bacterial suspensions in 100-well honeycomb plates. The final concentration of the tested extract ranged from 0.156 to 20 mg/mL. The plates were incubated for 24 h in a Bioscreen cell analyzer (Growth Curve Oy, Helsinki, Finland) at 37 °C, with continuous moderate shaking. Optical density (OD 600 nm) was measured every 1 h. Additionally, a positive control of tested bacterial growth and control of the medium sterility was prepared. The MIC value of the tested extract was determined as the lowest concentration that inhibited >90% of the microbial growth after 24 h of incubation at 37 °C in comparison with the positive control.

For the determination of MBC, a portion of liquid (5 µL) that exhibited no growth in the plate well was transferred to agar plates and incubated for 24 h at 37 °C. The lowest concentration that revealed no visible bacterial growth after subculturing was taken as the MBC. Positive and negative cultures were also prepared.

### 3.7. Cell Culture

Six cancer cell lines were used in the study. Breast adenocarcinoma (MCF-7) and colorectal adenocarcinoma (Caco-2; HT-29) cell lines were purchased from the Sigma-Aldrich company (ECACC, Steinheim, Germany). Melanoma (SK-Mel-29), glioblastoma (U87MG), and astrocytoma (U251MG) cell lines were obtained from the collection of the Nencki Institute of Experimental Biology, Polish Academy of Sciences, Warsaw, Poland. All cell lines were cultured at 37 °C with 5% CO_2_, in Dulbecco’s Modified Eagle Medium (DMEM) or McCoy’s 5A Medium, supplemented with fetal bovine serum (FBS) and antibiotics (penicillin/streptomycin). Cells were grown in an incubator (Thermo Heracell 150, Geneo Bio TechProducts, Bremen, Germany).

### 3.8. Cell Viability Assay

A CellTiter 96^®^ AQueous Non-Radioactive Cell Proliferation Assay (Promega) was used to determine cell viability. Cells were seeded into 96-well microplates at a density of 8–10 × 10^3^ cells per well and then incubated for 24 h at 37 °C to promote cell adhesion. The extract of *J. regia* flowers was dissolved in water and diluted in medium. Cells were exposed with medium at five concentrations of 10–750 µg extract/mL. After 24, 48, and 72 h of incubation at 37 °C, 20 µL of MTS solution was added to each well and they were stored in an incubator for one hour. The absorbance was measured at 490 nm with a microplate reader (Sunrise Microplate Reader Remote-Elisa Assays, Tecan Trading AG, Switzerland) and cell viability was estimated as a percentage relative to control (untreated cells). The results were expressed as the IC_50_.

### 3.9. Determination of Polyphenol Profile by UPLC-PDA-MS/MS

Determination of polyphenolic compounds was carried out using the UPLC apparatus equipped with a binary pump, column and sample manager, photodiode array detector (PDA), and tandem quadrupole mass spectrometer (TQD) with electrospray ionization (ESI) source working in negative mode (Waters, Milford, MA, USA) according to the method of Żurek et al. (2021) [39]. Separation was performed using the UPLC BEH C18 column (1.7 µm, 100 mm × 2.1 mm, Waters) at 50 °C, at an isocratic flow rate of 0.35 mL/min. The injection volume of the samples was 5 µL. The mobile phase consisted of water (solvent A) and 40% acetonitrile in water, *v*/*v* (solvent B). The following TQD parameters were used: capillary voltage of 3500 V; con voltage of 30 V; con gas flow 100 L/h; source temperature 120 °C; desolvation temperature 350 °C; and desolvation gas flow rate of 800 L/h. Polyphenolic identification and quantitative analyses were performed on the basis of the mass-to-charge ratio, retention time, specific PDA spectra, fragment ions, and comparison of data obtained with literature findings and commercial standards. The results were expressed as mg/100 g d.w.

The method was validated for parameters such as linearity, accuracy (relative error, RE), limit of detection (LOD), limit of quantification (LOQ), and precision (relative standard deviation, RSD). Quantification was determined by external standard calibration. Stock standard solutions of the polyphenols were prepared with methanol. Six calibrators of each standard were prepared by dilution of stock solutions, and the calibration curve was generated by plotting the peak area ratio of the polyphenol versus the nominal concentration ranging from 0.05 to 5 mg mL^−1^ (R^2^ ≤ 0.999). The regression equation was obtained by weighted (1/c2) least-squares linear regression. The LOD was determined as a signal-to-noise ratio (S/N) of 3:1, and the LOQ was determined as an S/N of >10. An acceptable RE within ±20% and an RSD not exceeding 20% should be obtained.

### 3.10. Statistical Analysis

All experiments were performed in triplicate. Results were expressed as the mean ± standard deviation (SD). Statistical analysis, including one-way analysis of variance, was performed using Statistica 13.3 (StatSoft, Kraków, Poland). Statistically significant differences (*p* = 0.05) between the mean values were assessed by Duncan’s test. Additionally, between the parameters characterizing the properties of the flowers, the values of Pearson’s correlation coefficients were calculated, the significance of which was tested at the level of *p* = 0.01 and *p =* 0.05.

## 4. Conclusions

In conclusion, the results emerging from this study allow us to fill a gap in relation to the scientific knowledge about *J. regia* male flowers. This work confirms that walnut inflorescences are a valuable source of polyphenols. The high content of phenolics, mainly quercetin derivatives, is reflected in the high antioxidant capacity. Moreover, *J. regia* flower extract has shown antibacterial and antiproliferative activity. Thus, considering the excellent cultivation and great abundance of the species in almost all regions of the world, together with the significant pro-health properties of the extract, the flowers could be easily exploited as an excellent source of effective natural antioxidants and chemopreventives in a wide range of biotechnology products. In addition, further research for this valuable raw material includes, among others, in vivo analysis. This would enable the development of many effective therapeutic strategies using individual active compounds or mixtures isolated from the flowers of *J. regia*.

## Figures and Tables

**Table 1:** The contents of total phenolics (TP), flavonoids (TF), and proanthocyanidins (TPA) of *J. regia* male flower extracts.

	TP	TF	TPA
	(mg GAE/g d.w.)	(mg QE/g d.w.)	(mg CYE/g d.w.)
Extract of *J. regia* flowers	248.33 ± 2.33	111.01 ± 1.03	16.66 ± 0.70

Abbreviations: TP, total phenolic content; TF, total flavonoid content; TPA, total proanthocyanidin content; GAE, equivalent of gallic acid; QE, equivalent of quercetin; CYE, equivalent of cyanidin chloride; d.w., dry weight. Values are expressed as mean ± SD.

**Table 2:** Antioxidant activities of *J. regia* male flowers extracts and standards.

	DPPH˙	ChA	O^2●−^	OH^−^	CUPRAC
	IC_50_ (µg/mL)				(mmol TE/g)
Extract of *J. regia* flowers	22.34 ± 2.70	71.69 ± 0.02	147.06 ± 0.27	41.85 ± 0.09	3.33 ± 0.01
Ascorbic acid	5.0 ± 0.01	-	80.58 ± 1.16	-	62.19 ± 0.74
EDTA	-	16.19 ± 0.09	-	-	-
Quercetin	-	-	-	9.34 ± 0.01	-

Abbreviations: DPPH˙, 2,2-diphenyl-1-picrylhydrazyl radical scavenging activity assay; ChA, chelating ability of ferrous ions; O^2●−^, superoxide radical scavenging activity assay; OH^−^, hydroxyl radical scavenging activity assay; CUPRAC, copper ion reduction assay; IC_50_, half the maximum inhibitory concentration; TE, Trolox equivalent; EDTA, ethylenediaminetetraacetic acid. Ascorbic acid, EDTA, and quercetin were used as positive controls; (-) not tested. Values are expressed as mean ± SD.

**Table 3:** Antimicrobial activity of tested *J*. *regia* flower extract against Gram-positive and Gram-negative bacteria.

No.	Bacteria Strain	MIC	MBC
		(mg/mL)	
1	*Klebsiella pneumoniae*	20	20
2	*Salmonella enterica*	2.5	2.5
3	*Escherichia coli*	1.25	1.25
4	*Staphylococcus aureus*	0.3125	0.3125
5	*Listeria monocytogenes*	0.625	2.5
6	*Bacillus cereus*	0.625	0.625

Abbreviations: MIC, minimum inhibitory concentration; MBC, minimum bactericidal concentration.

**Table 4:** IC_50_ (µg/mL) values for the examined *J. regia* flower extracts on the viability of six human cancer cell lines after 24, 48, and 72 h of incubation.

No.	Cell Line	IC_50_ (µg/mL)		
		Time		
		24 h	48 h	72 h
1	MCF-7	132.35 ± 8.73	208.80 ± 3.42	194.22 ± 8.26
2	SK-MEL-29	593.62 ± 16.88	618.74 ± 10.70	706.21 ± 11.01
3	Caco-2	579.99 ± 13.36	443.49 ± 19.83	222.59 ± 10.04
4	HT-29	349.12 ± 9.20	401.83 ± 6.07	371.73 ± 9.03
5	U87MG	200.55 ± 11.01	200.42 ± 4.98	220.05 ± 12.06
6	U251MG	195.79 ± 8.57	206.95 ± 9.00	199.26 ± 9.10

Abbreviations: MCF-7, breast adenocarcinoma cells; SK-Mel-29, melanoma cells; Caco-2 and HT-29, colorectal adenocarcinoma cells; U87MG, glioblastoma cells; U251MG, astrocytoma cells; IC_50_, half maximum inhibitory concentration. Values are expressed as mean ± SD from three independent experiments.

**Table 5:** The phenols identified (tentatively) in *J. regia* male flowers by means of UPLC/PDA/MS/MS.

No.	Rt	UV–Vis	[M-H]^−^	[M-H]^−^ MS/MS	Extract of *J. regia* Flowers	Compounds
	(min)	(nm)	(*m/z*)	(*m/z*)	(mg/100 g d.w.)	
1	2.18	299sh, 324	353	191, 179	12.13	3-*O*-Caffeoylquinic acid
2	2.35	299sh, 324	353	191, 179	532.66	5-*O*-Caffeoylquinic acid
3	2.55	299sh, 324	341	179	107.00	Caffeic acid glucoside
4	2.70	299sh, 324	341	179	58.82	Caffeic acid glucoside
5	2.88	310	337	163, 119	207.17	Coumaroylquinic acid
6	2.98	299sh, 322	353	191, 179	94.59	4-*O*-Caffeoylquinic acid
7	3.49	257, 317	339	179	74.76	Juglanoside B isomer
8	3.54	305, 325, 338	499	337, 163	627.49	3-*O*-caffeoyl-5-*O*-*p*-coumaroylquinic acid
9	3.61	258, 339	355	175	14.25	Juglanoside D isomer
10	3.67	309	337	163, 119	20.06	Coumaroyloquinic acid
11	3.83	260, 350	355	175	12.13	Juglanoside D isomer
12	3.95	255, 352	625	463, 301	815.24	Quercetin diglucoside
13	4.39	264, 341	609	447, 285	28.71	Kaemferol diglucoside
14	4.67	255, 352	463	301	870.65	Quercetin 3-*O*-glucoside
15	4.74	262, 331	491	329	294.74	4′,5,7-Trihydroxy-3,6-dimethoxyflavone-7-*O*-beta-d-glucopyranoside
16	4.99	255, 352	433	301	40.98	Quercetin pentoside
17	5.11	264, 357	447	285	169.52	Kaempherol 3-*O*-glucoside
18	5.19	255, 352	433	301	44.09	Quercetin pentoside
19	5.40	255, 360	447	301	99.04	Quercetin rhamnoside
20	5.61	272, 353	433	271	21.11	Naringenin 7-*O*-glucoside
21	6.25	288sh, 324	501	179	188.64	Unidentified caffeic derivative
22	6.66	327	517	335, 179	35.11	Caffeic acid glucoside glucuronide
23	7.28	299sh, 327	515	353	13.48	3,4-*O*-dicaffeoylquinic acid
24	7.41	288sh, 324	501	179	14.36	Unidentified caffeic derivative
Total					4396.73	

Abbreviations: Rt, retention time; UV–Vis, ultraviolet–visible; [M-H]^−^, negative ion values; *m*/*z*, mass-to-charge ratio; d.w., dry weight.

## Data Availability

Not applicable.

## Supplementary Materials

The following attachments are available online at https://www.mdpi.com/article/10.3390/molecules27092762/s1, Figure S1: Antioxidant activities of *J. regia* male flower extracts (10–750 µg/mL); Figure S2: UPLC chromatogram of *J. regia* male flower extracts.

## Sample Availability

Samples of the compounds are available from the authors.
