# Supplementary material for: Phenolic Profile and Antioxidant, Antibacterial, and Antiproliferative Activity of Juglans regia L. Male Flowers
Source: Molecules. 2022 Apr 26;27(9):2762. doi: 10.3390/molecules27092762 (PMC9101975; doi:10.3390/molecules27092762)
Supplement: Supplementary file 1 [file molecules-27-02762-s001.zip › molecules-1683775-supplementary.pdf]

molecules

MDPI

Supplementary material

Phenolic profile and antioxidant, antibacterial, antiproliferative activity of *Juglans regia* L. male flowers

Natalia Żurek [1], Agata Pawłowska [1], Karolina Pycia [1], Dorota Grabek-Lejko [2] and Ireneusz Kapusta [1,*]

[1] Department Food Technology and Human Nutrition, Institute of Food Technology and Nutrition, University of Rzeszow, 4 Zelwerowicza St., 35-601 Rzeszow, Poland; nzurek@ur.edu.pl (N.Ż.); agpawlowska@ur.edu.pl (A.P.); kpycia@ur.edu.pl (K.P.); ikapusta@ur.edu.pl (I.K.)
[2] Department of Bioenergetics, Food Analysis and Microbiology, Institute of Food Technology and Nutrition, University of Rzeszow, 4 Zelwerowicza St., 35-601 Rzeszow, Poland; dgrabek@ur.edu.pl (D.G-L.)
* Correspondence: ikapusta@ur.edu.pl; Tel.: +48-17-785-5238

Content:

Supplementary Figure S1: Antioxidant activities of *J. regia* male flowers extracts (10-750 μg/ml).

Supplementary Figure S2: UPLC chromatogram of *J. regia* male flowers extracts and UV-Vis and MS spectra of identified compounds.

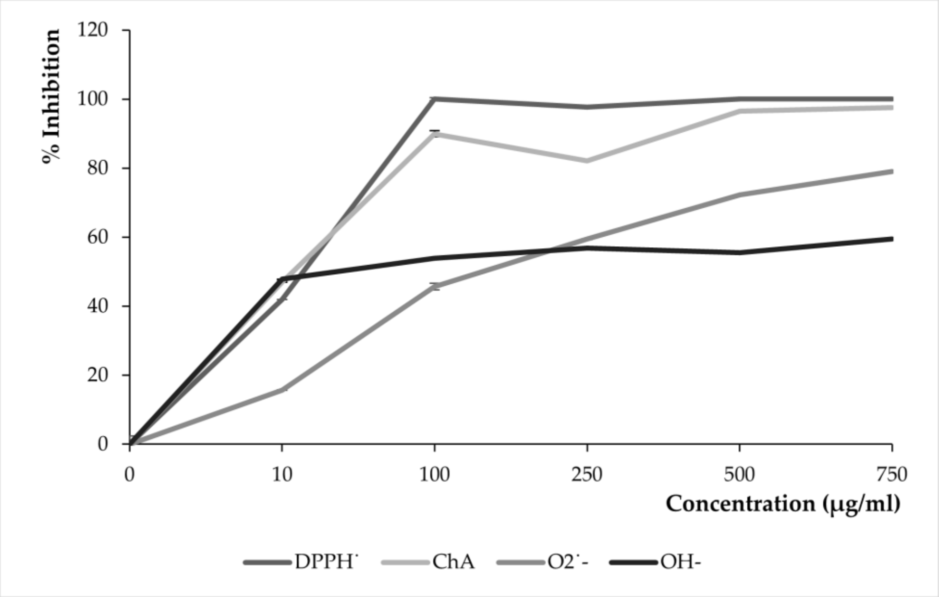

Figure S1. Antioxidant activities of *J. regia* male flowers extracts (10-750 μg/ml). Graphs represent mean values ±SD from three independent experiments. Abbreviations: DPPH˙, 2,2-diphenyl-1-picrylhydrazyl radical scavenging activity assay; ChA, chelating ability of ferrous ion; $O^{2\cdot-}$, superoxide radical **s**cavenging **a**ctivity **assay**; OH^-, hydroxyl radical scavenging activity assay; CUPRAC, copper ion reduction assay.

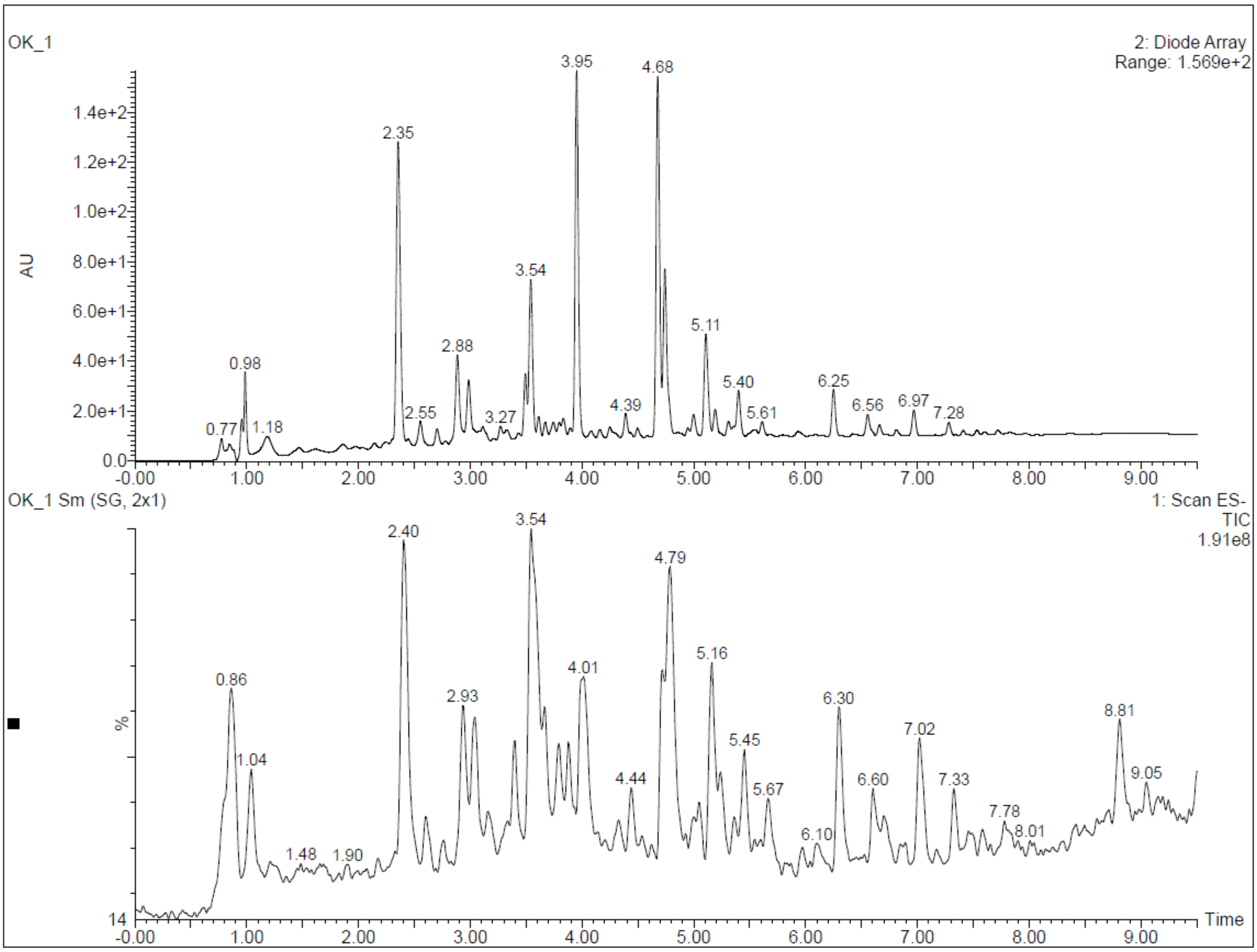

Figure S2: UPLC chromatogram of *J. regia* male flowers extracts and UV-Vis and MS spectra of identified compounds.
