# Supplementary figures and images for: Phenolic Profile and Antioxidant, Antibacterial, and Antiproliferative Activity of Juglans regia L. Male Flowers
Source: Molecules. 2022 Apr 26;27(9):2762. doi: 10.3390/molecules27092762 (PMC9101975; doi:10.3390/molecules27092762)

OK\_1

2: Diode Array  
Range: 1.569e+2

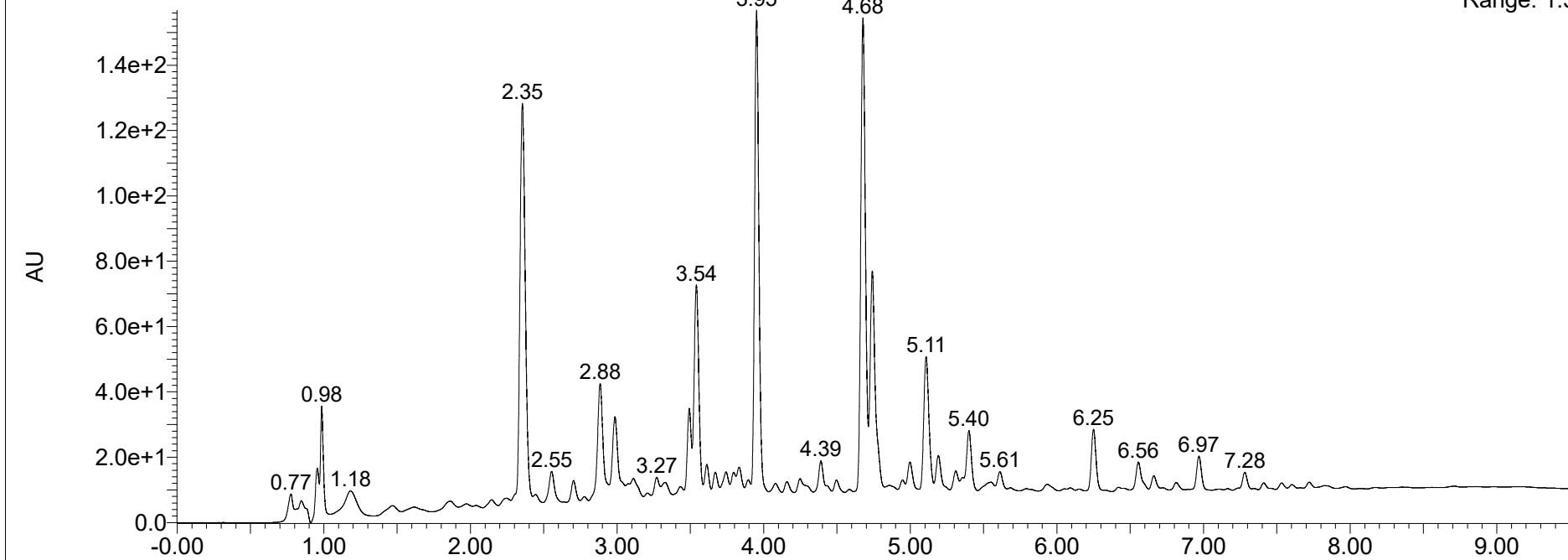

OK\_1 Sm (SG, 2x1)

1: Scan ES-  
TIC  
1.91e8

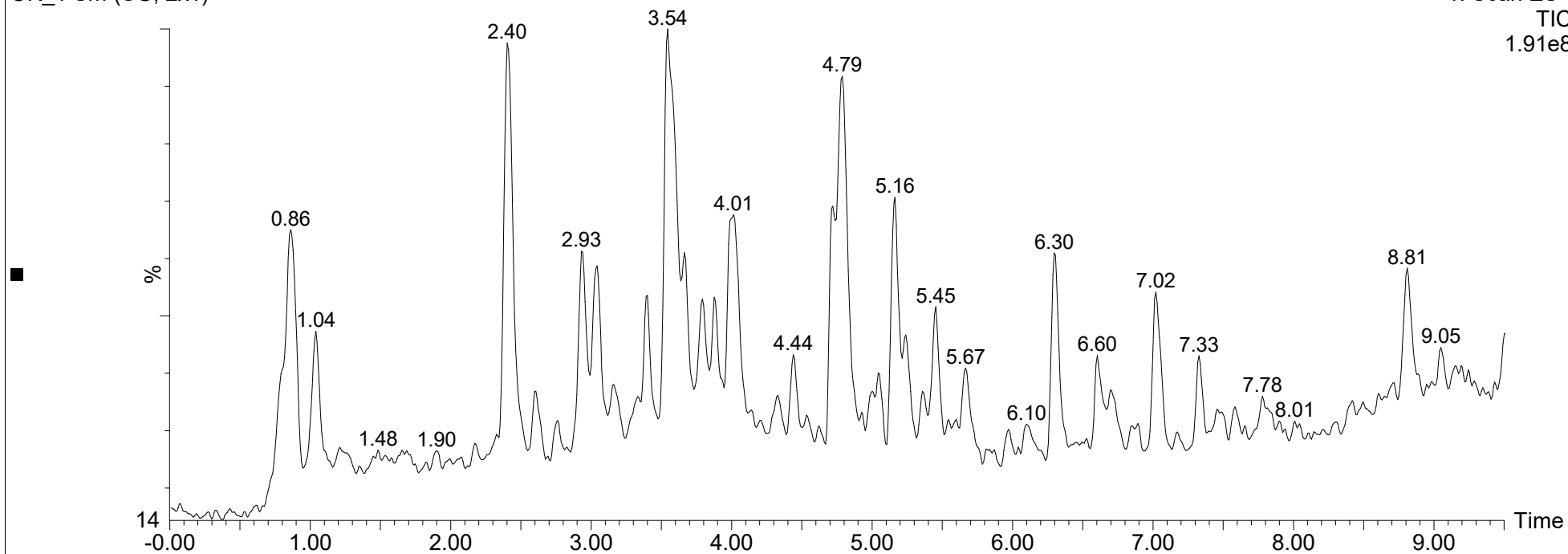

Supplement: Supplementary file 1 [file molecules-27-02762-s001.zip › Chromatogram.pdf]

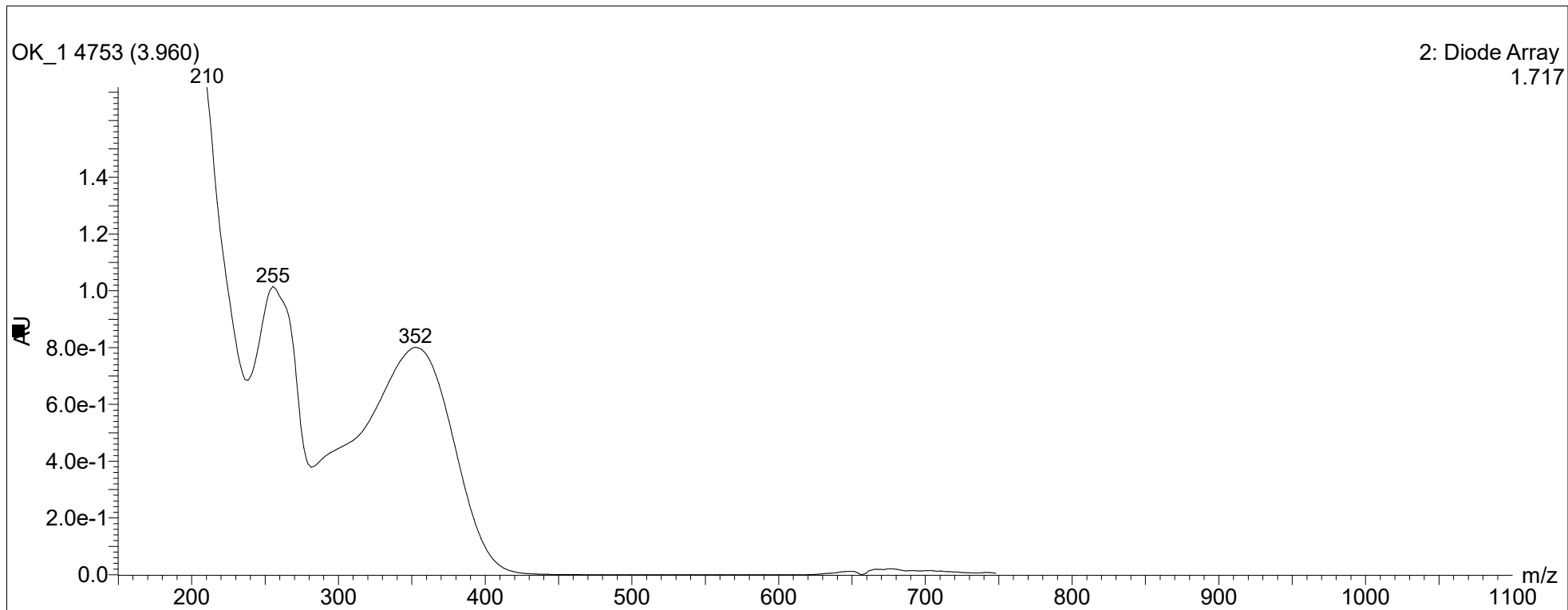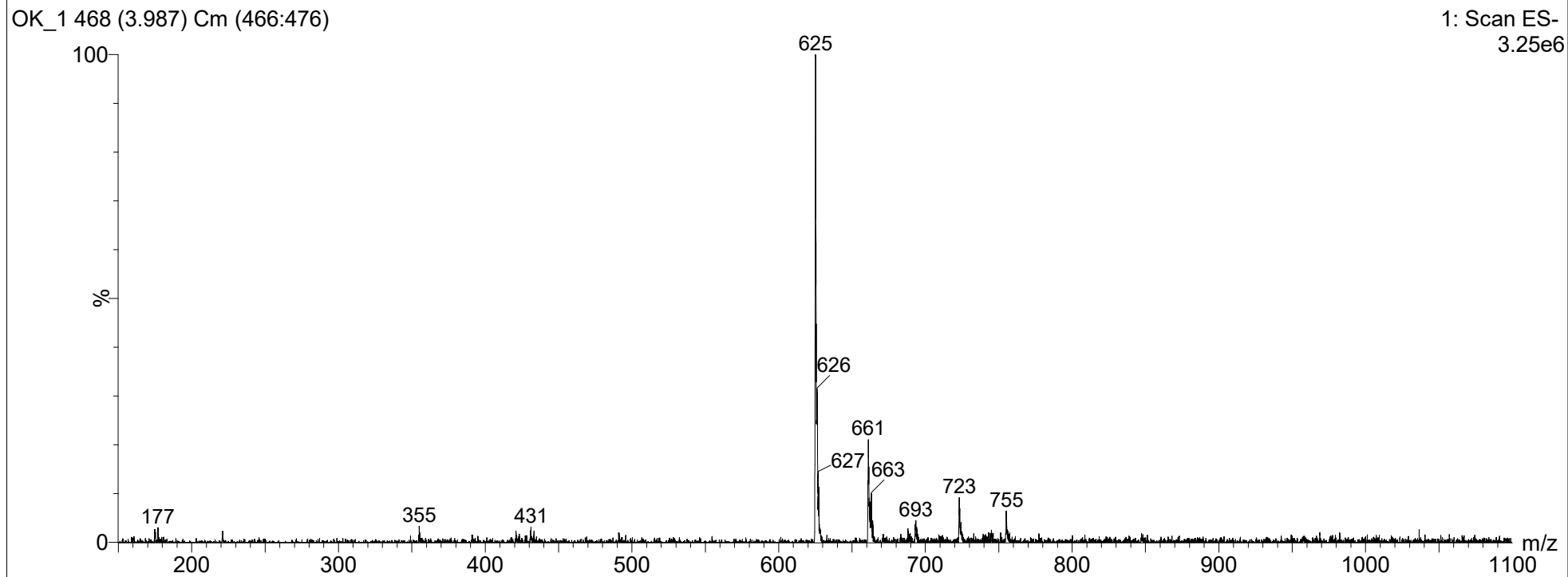

Supplement: Supplementary file 1 [file molecules-27-02762-s001.zip › Compound 12.pdf]

OK\_1 5269 (4.390)

2: Diode Array  
4.942e-1

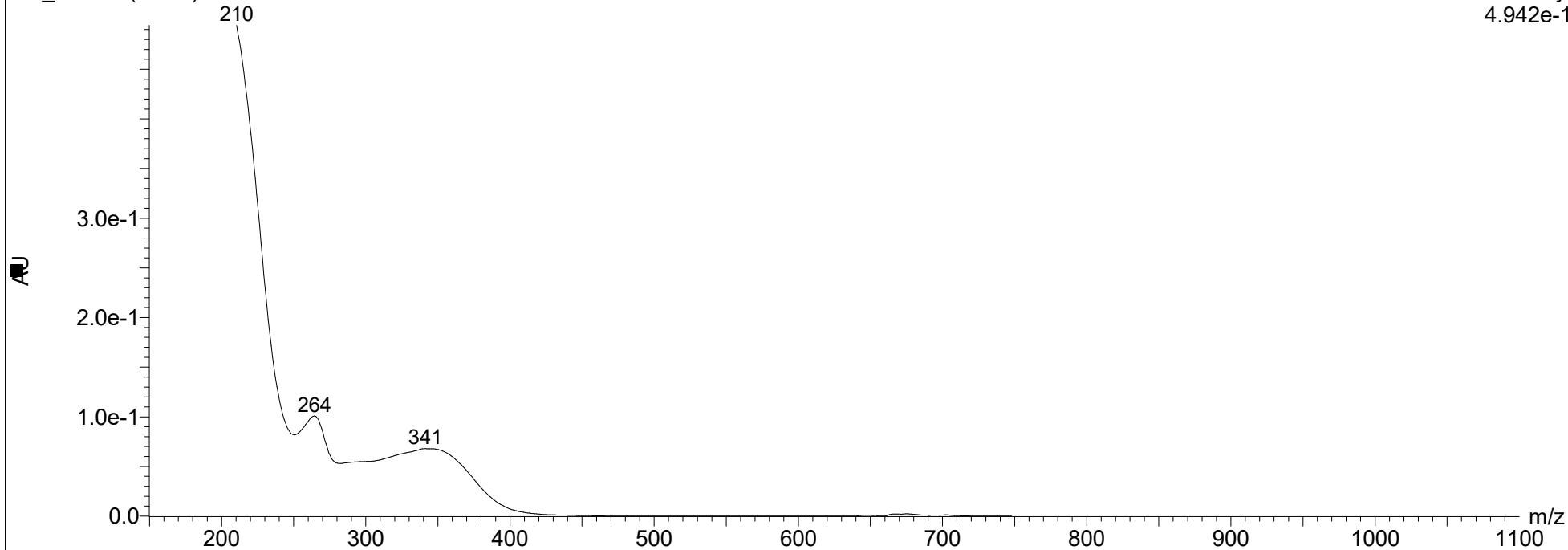

OK\_1 522 (4.447) Cm (518:525)

1: Scan ES-  
4.87e5

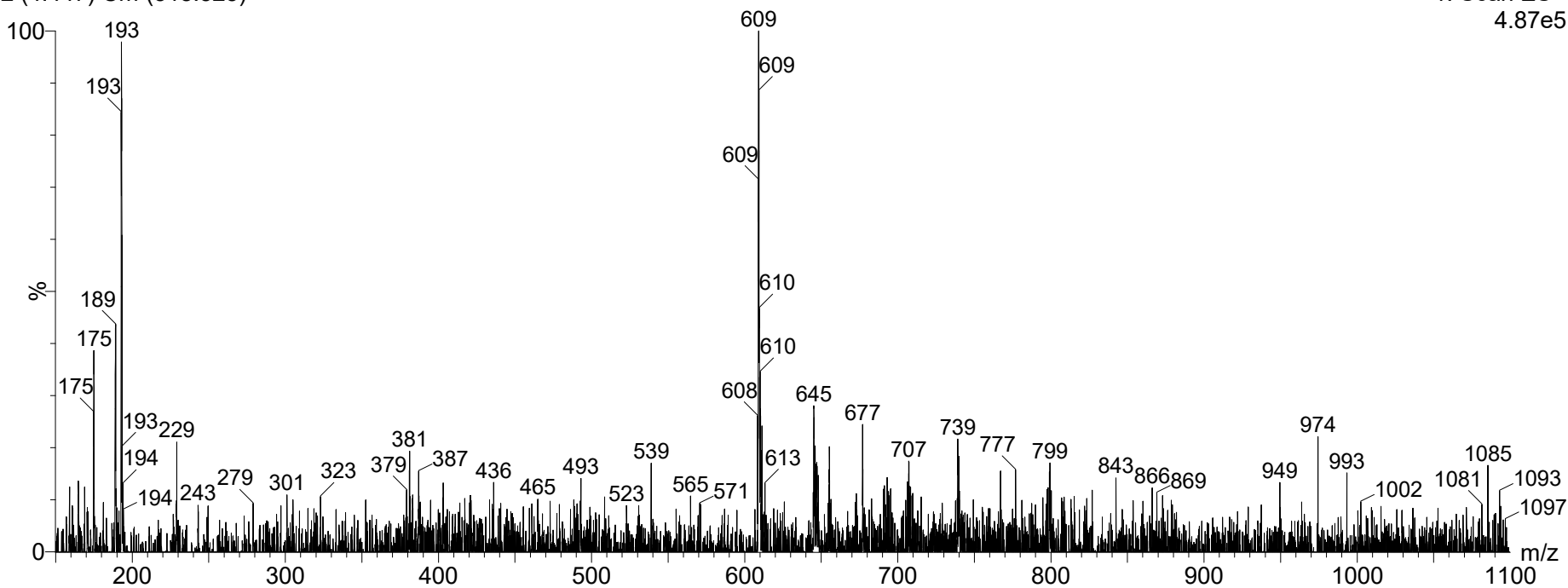

Supplement: Supplementary file 1 [file molecules-27-02762-s001.zip › Compound 13.pdf]

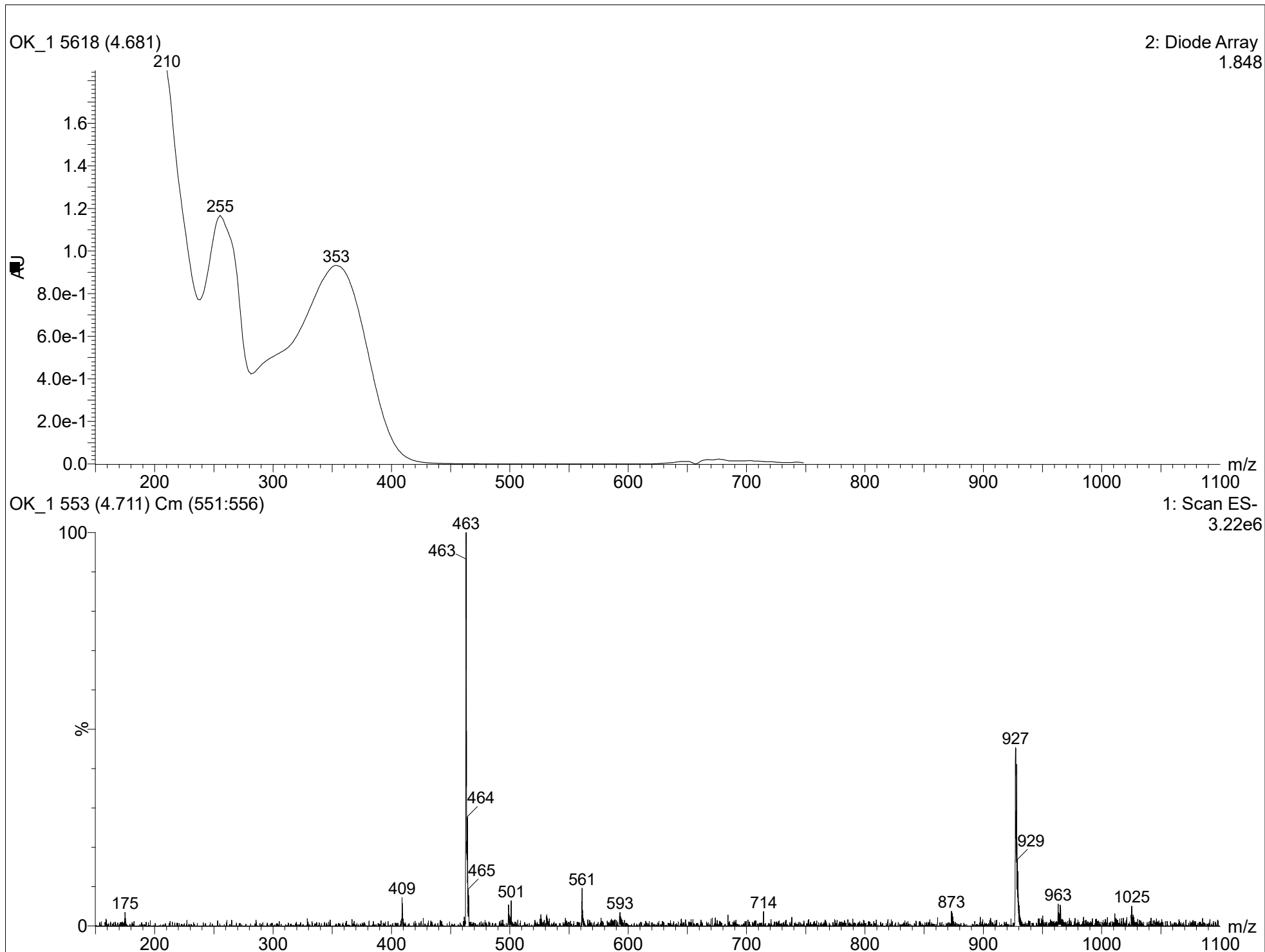

Supplement: Supplementary file 1 [file molecules-27-02762-s001.zip › Compound 14.pdf]

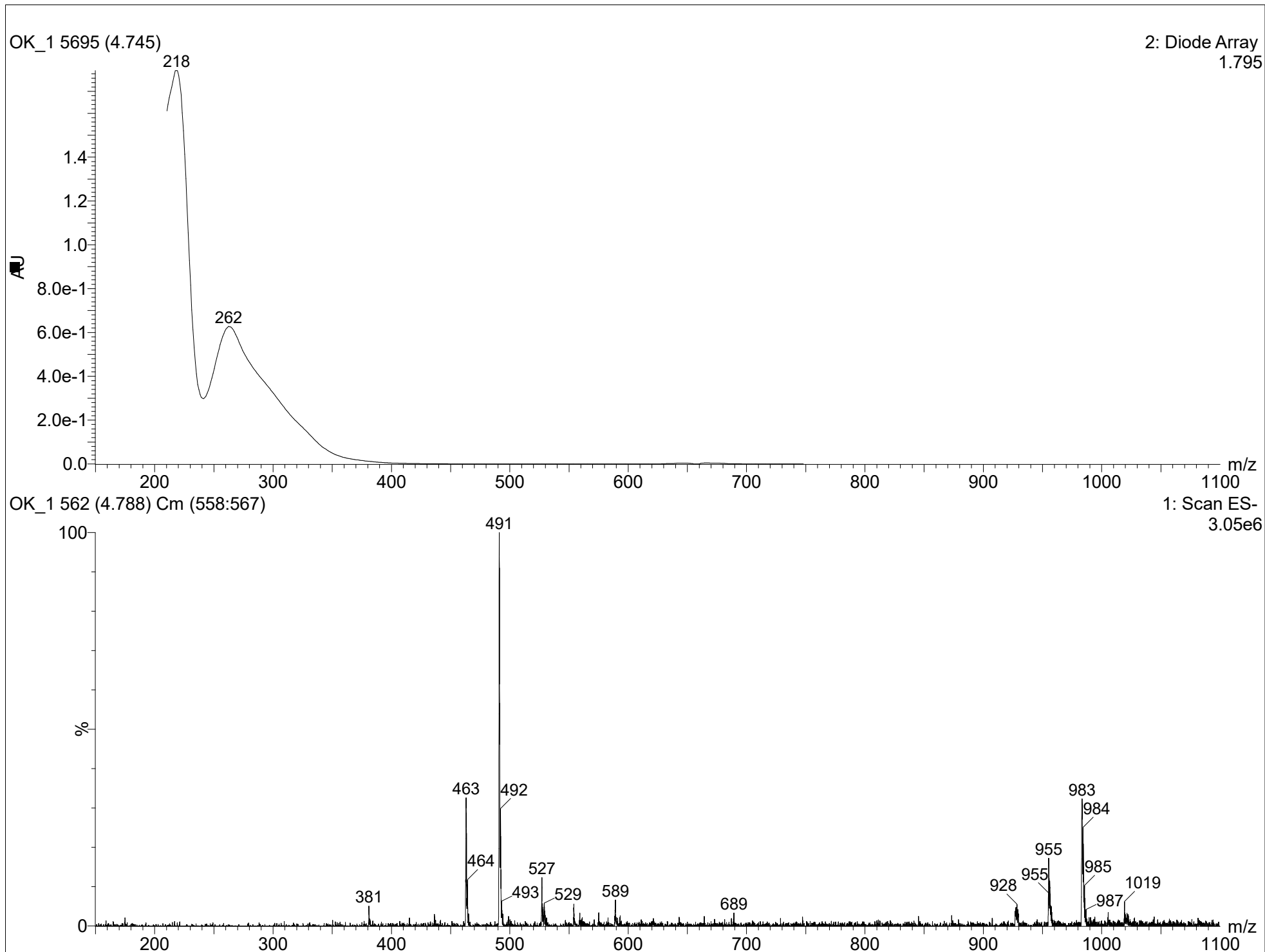

Supplement: Supplementary file 1 [file molecules-27-02762-s001.zip › Compound 15.pdf]

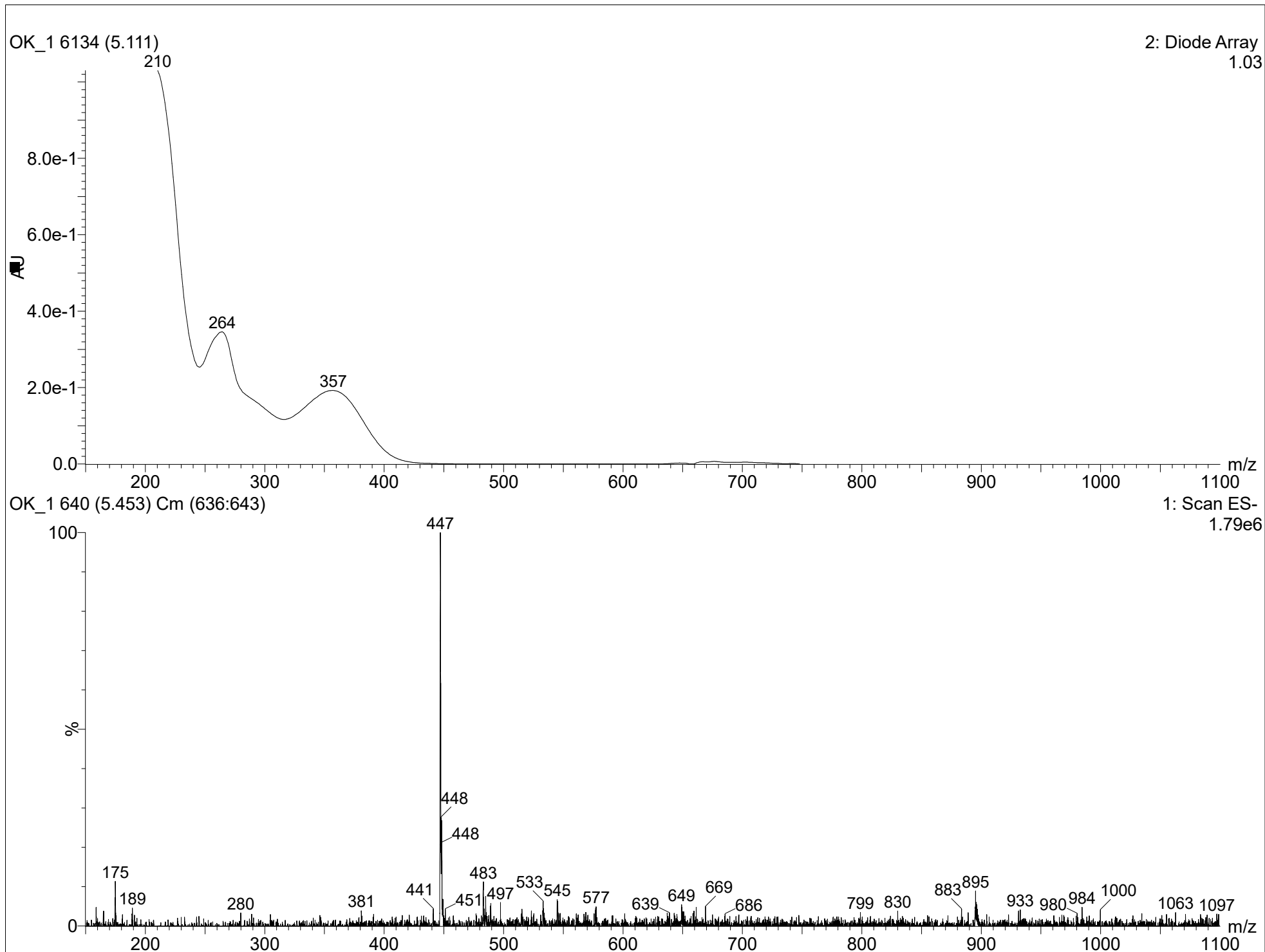

Supplement: Supplementary file 1 [file molecules-27-02762-s001.zip › Compound 17.pdf]

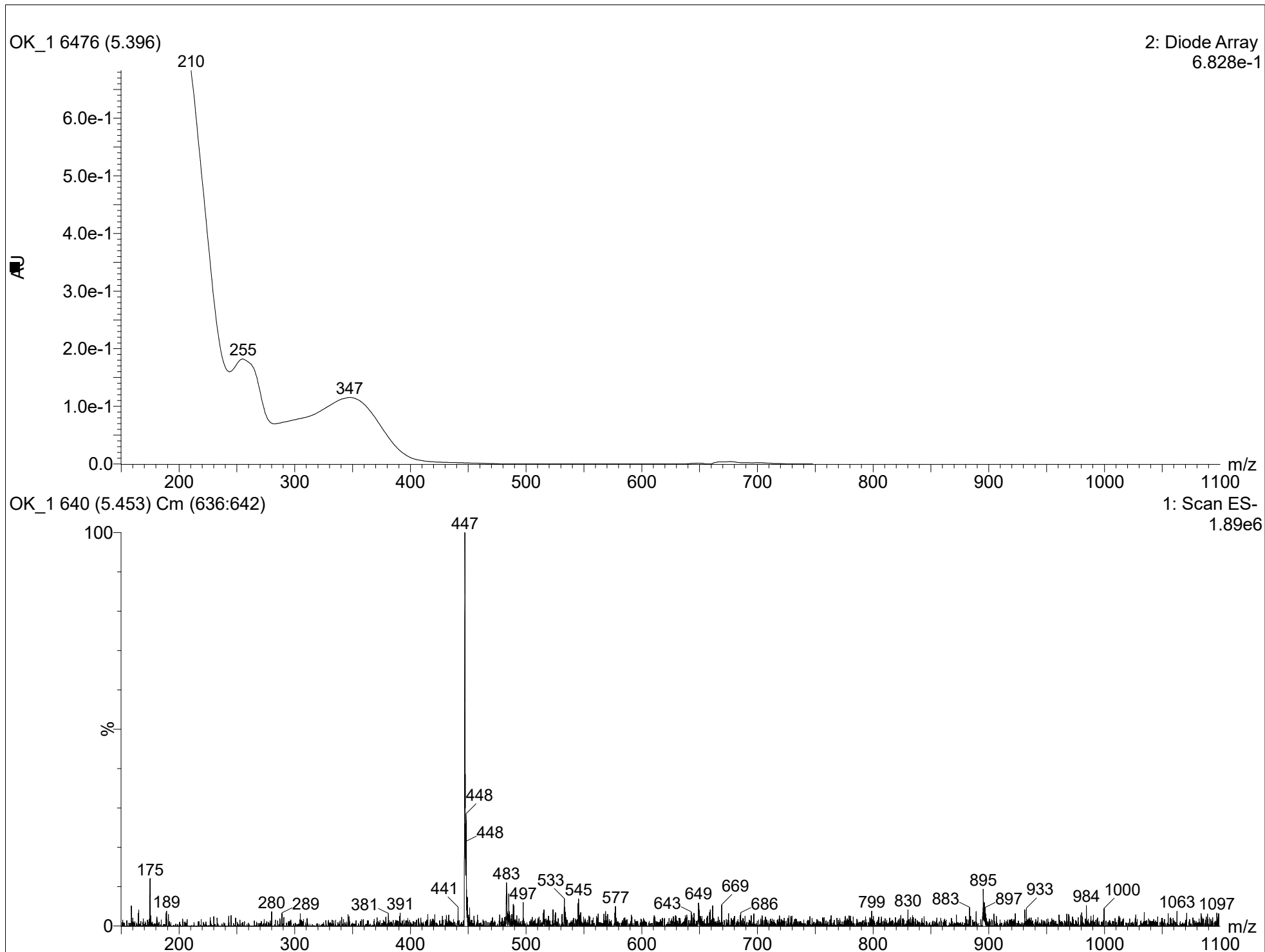

Supplement: Supplementary file 1 [file molecules-27-02762-s001.zip › Compound 19.pdf]

OK\_1 6741 (5.617)

2: Diode Array  
5.525e-1

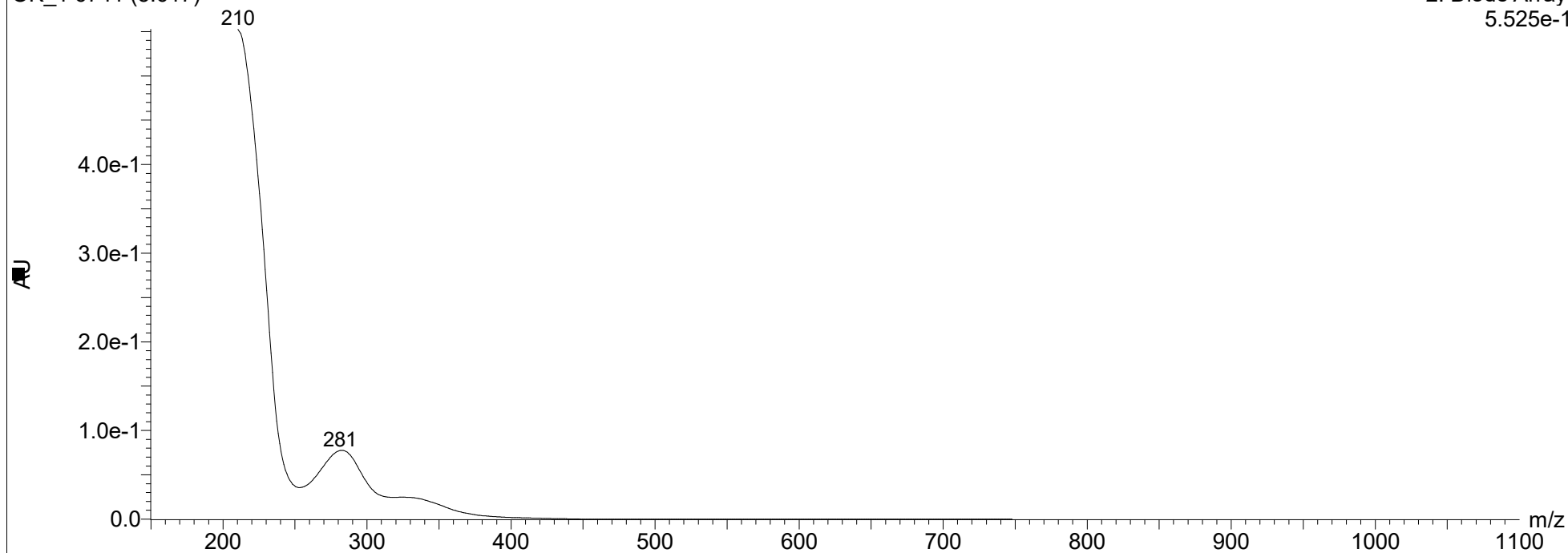

OK\_1 666 (5.674) Cm (662:668)

1: Scan ES-  
1.09e6

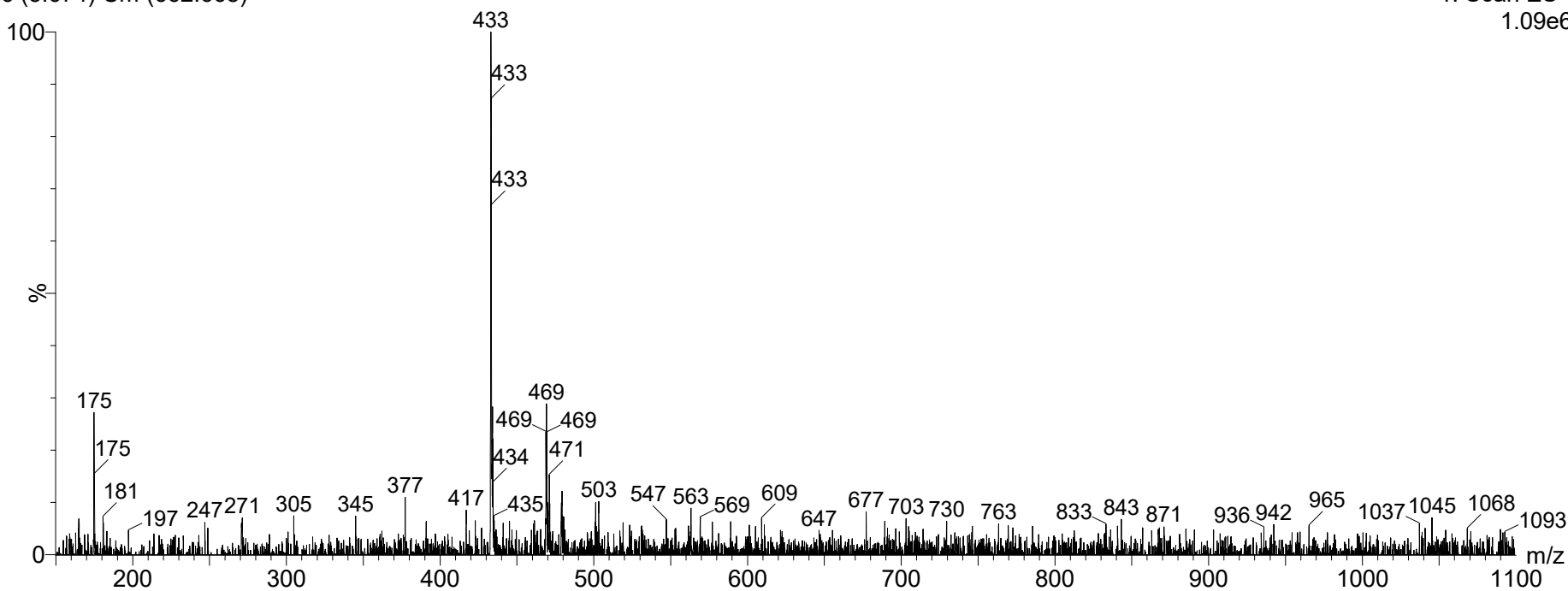

Supplement: Supplementary file 1 [file molecules-27-02762-s001.zip › Compound 20.pdf]

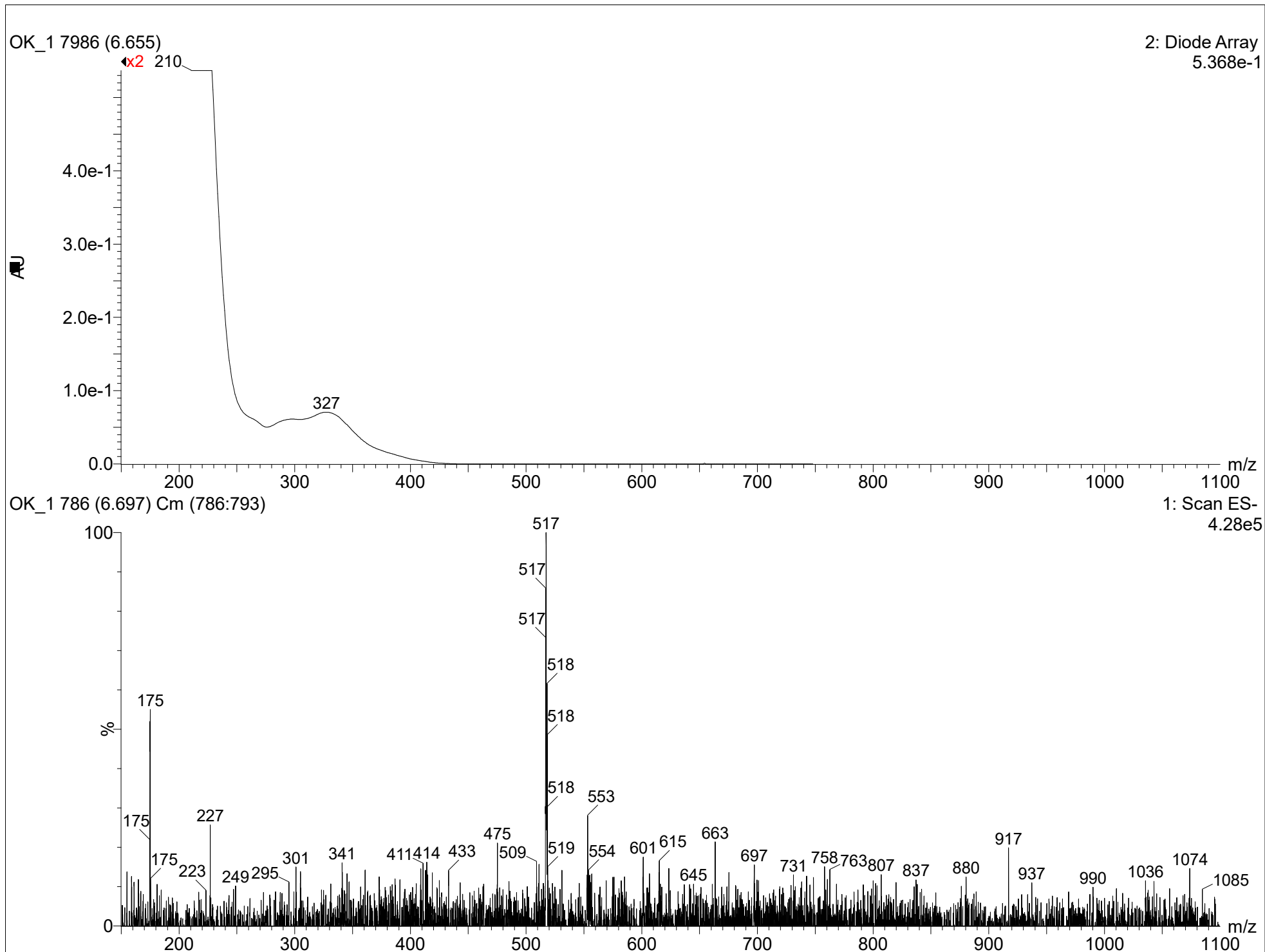

Supplement: Supplementary file 1 [file molecules-27-02762-s001.zip › Compound 22.pdf]

OK\_1 860 (7.327) Cm (858:865)

1: Scan ES-  
8.11e5

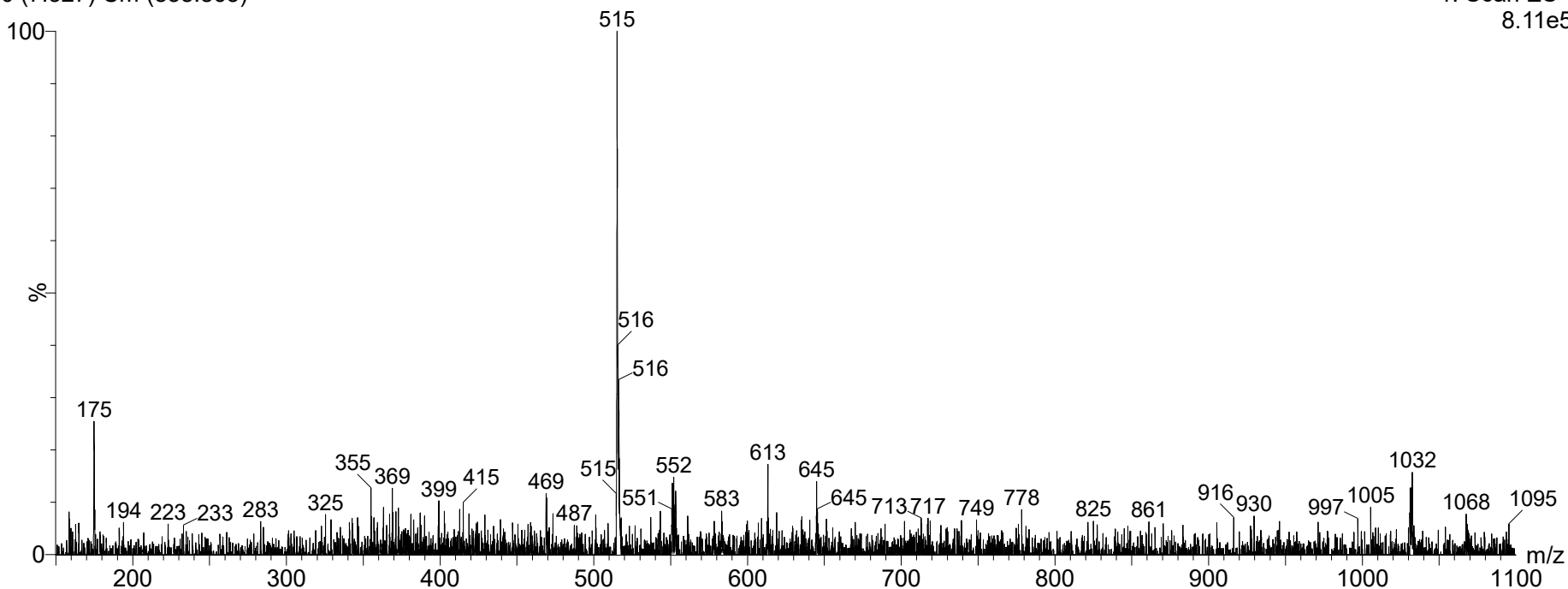

OK\_1 8742 (7.285)

2: Diode Array  
5.773e-1

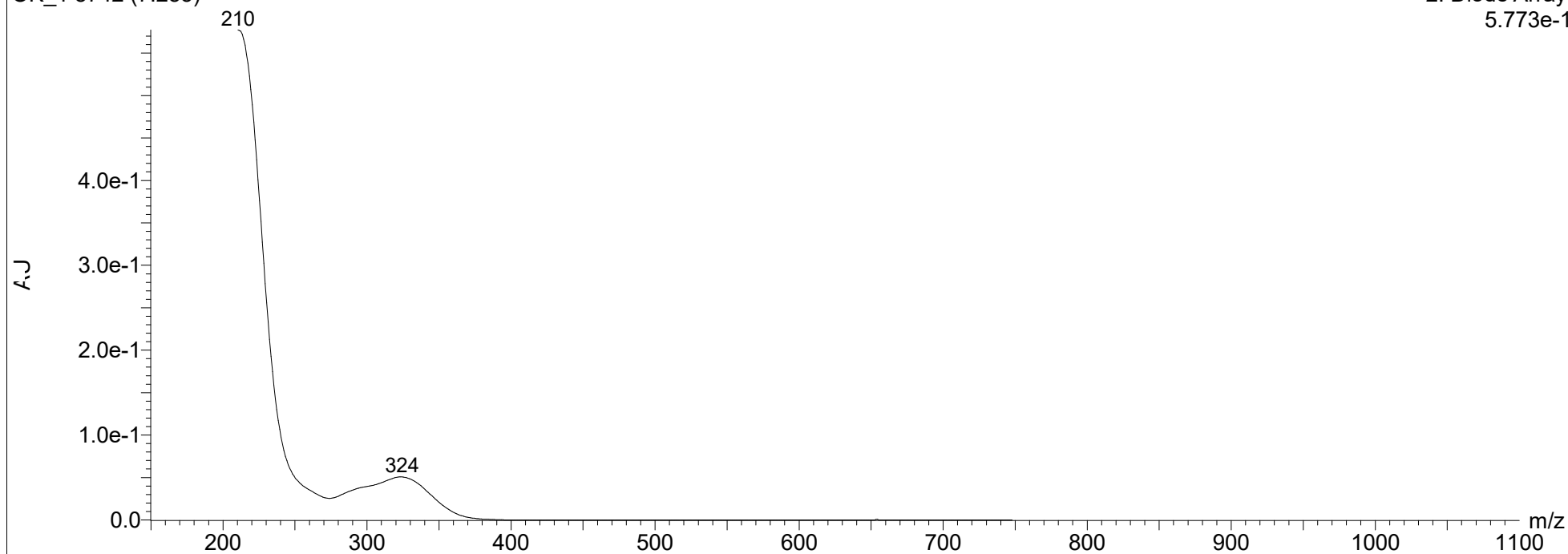

Supplement: Supplementary file 1 [file molecules-27-02762-s001.zip › Compound 23.pdf]

OK\_1 4191 (3.492)

2: Diode Array  
9.075e-1

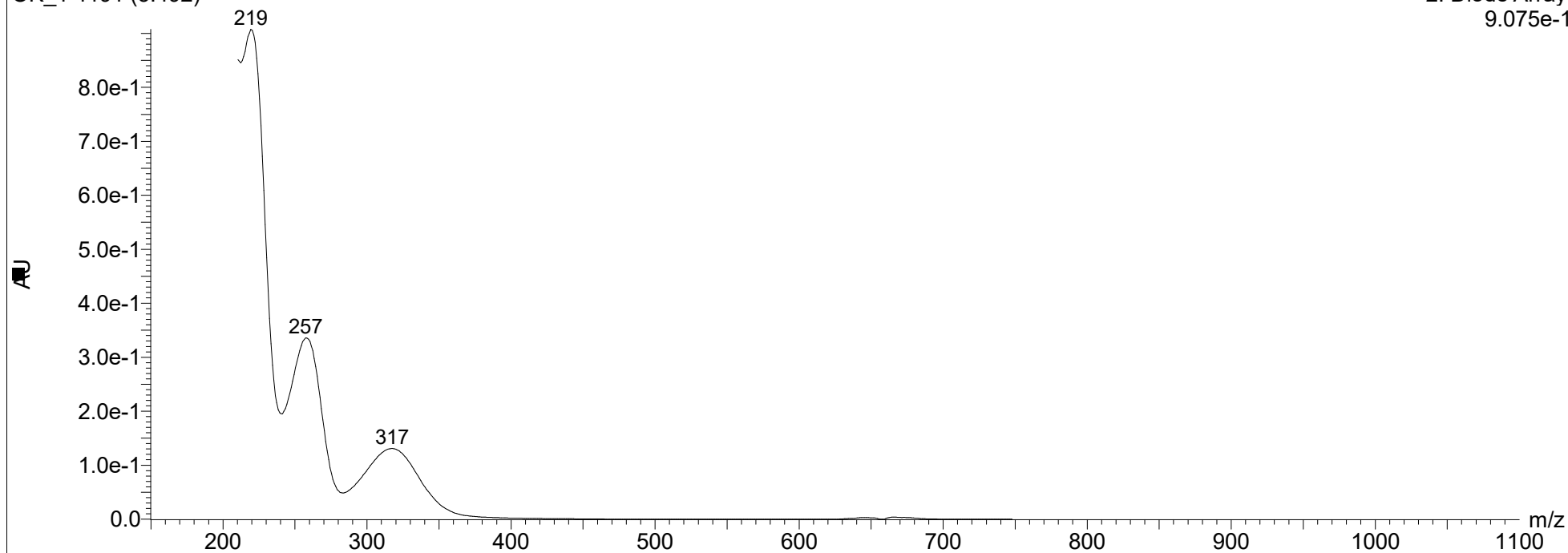

OK\_1 416 (3.544) Cm (414:419)

1: Scan ES-  
2.98e6

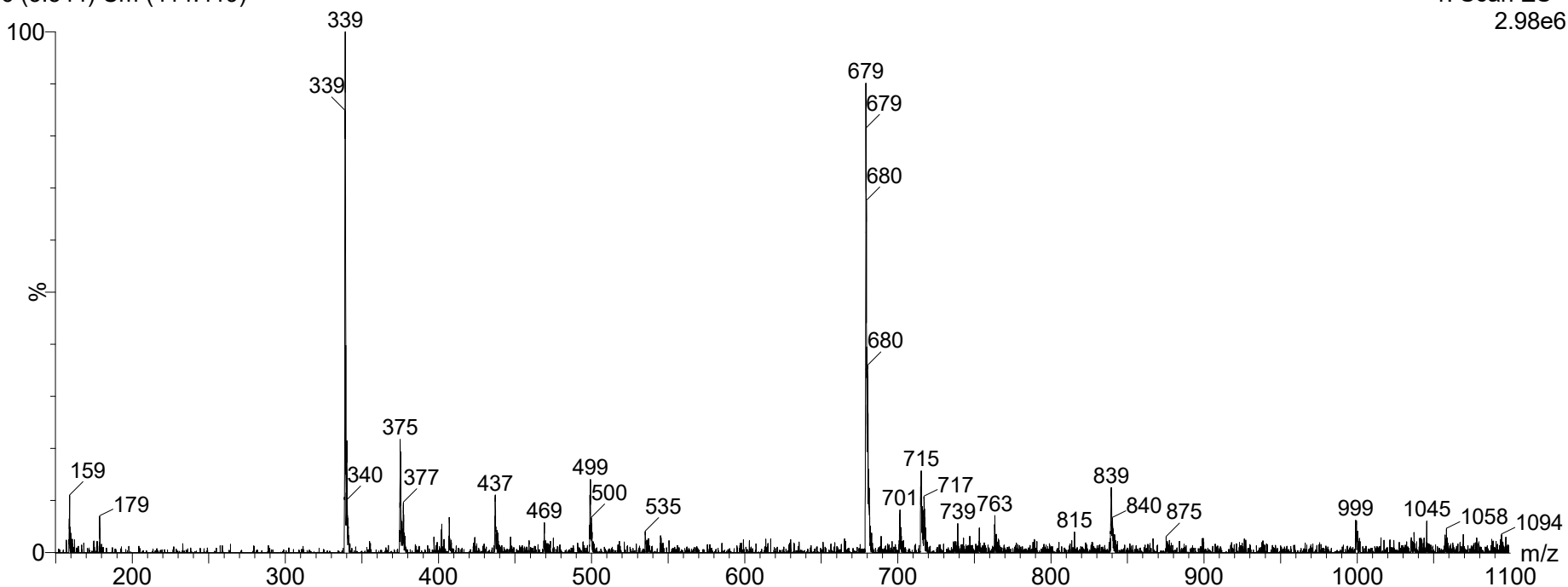

Supplement: Supplementary file 1 [file molecules-27-02762-s001.zip › Compound 7.pdf]

OK\_1 4262 (3.551)

2: Diode Array  
1.939

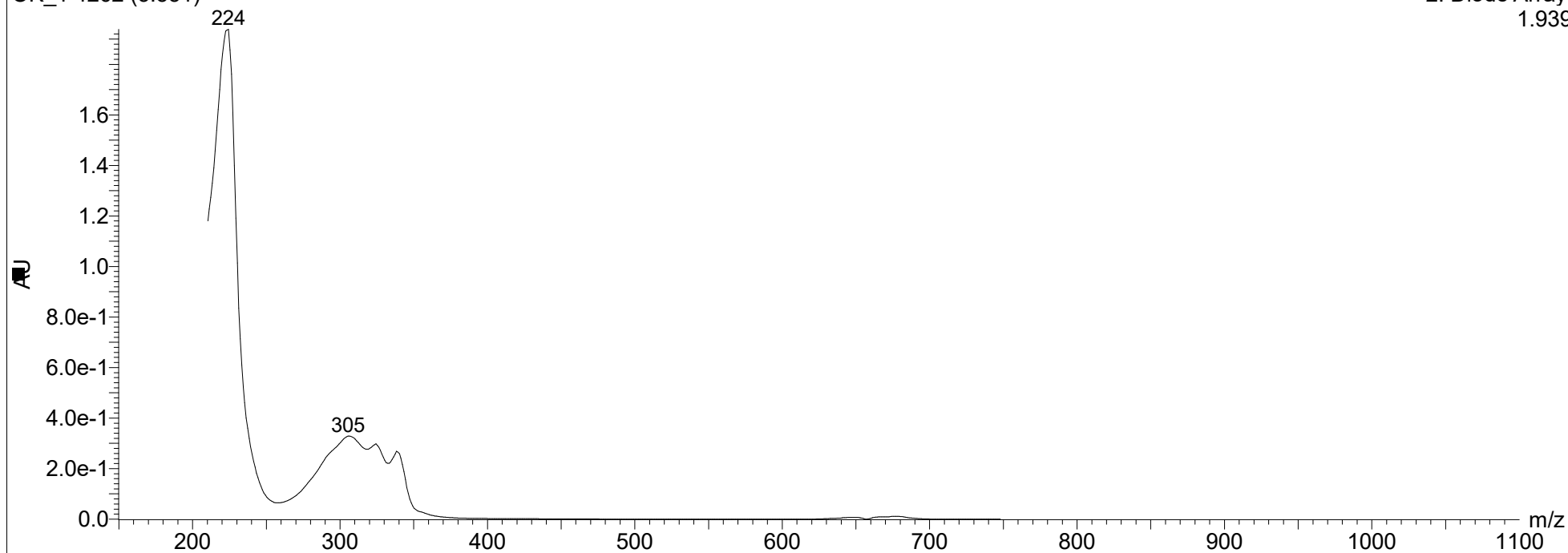

OK\_1 420 (3.578) Cm (420:428)

1: Scan ES-  
2.07e6

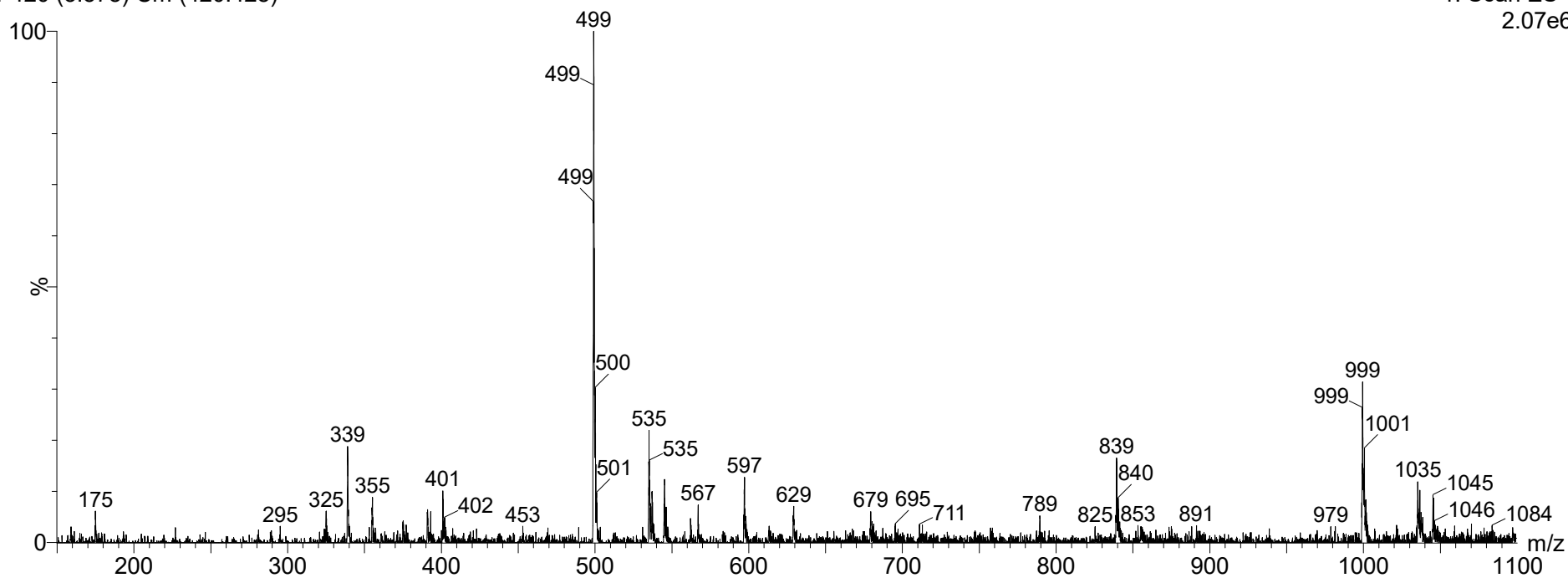

Supplement: Supplementary file 1 [file molecules-27-02762-s001.zip › Compound 8.pdf]

OK\_1 2835 (2.362)

2: Diode Array  
1.161

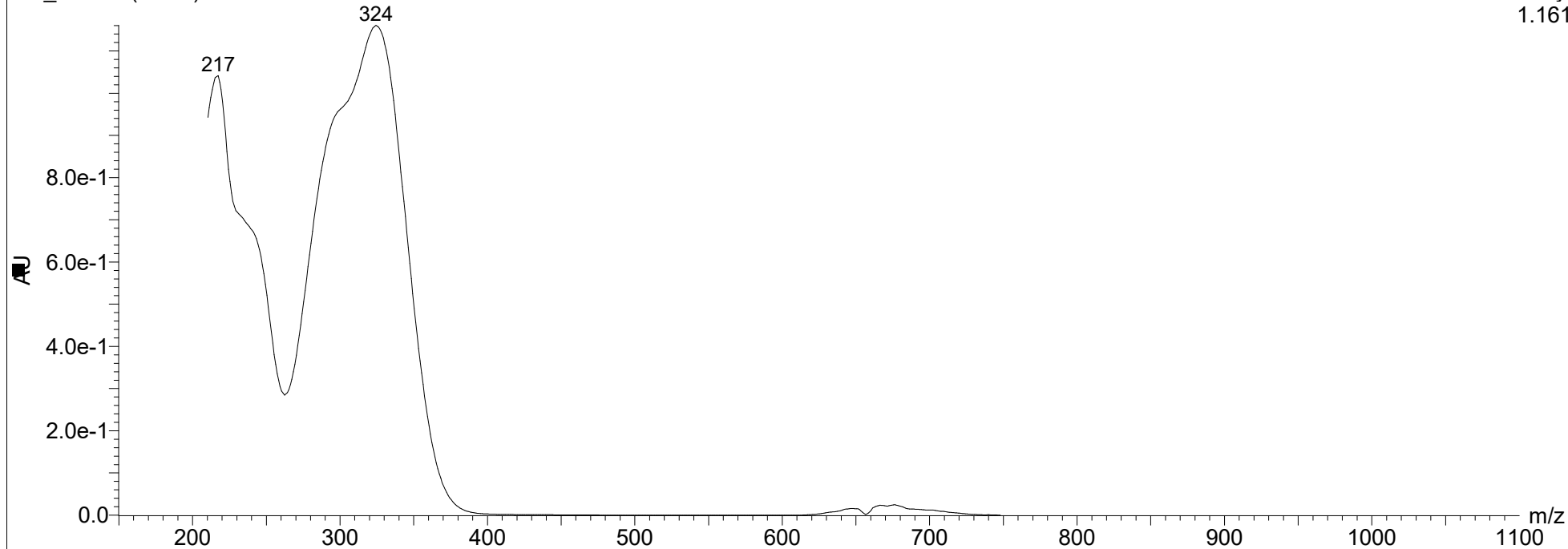

OK\_1 282 (2.403) Cm (276:289)

1: Scan ES-  
3.23e6

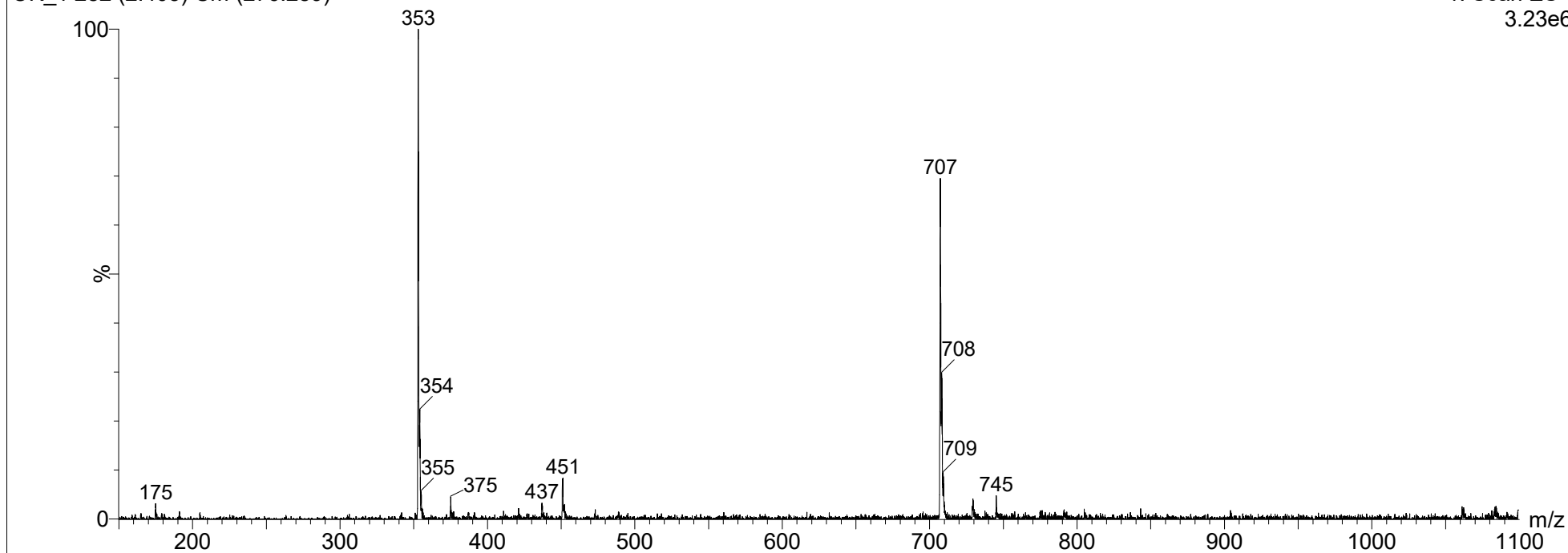

Supplement: Supplementary file 1 [file molecules-27-02762-s001.zip › Compounds 1,2,6.pdf]

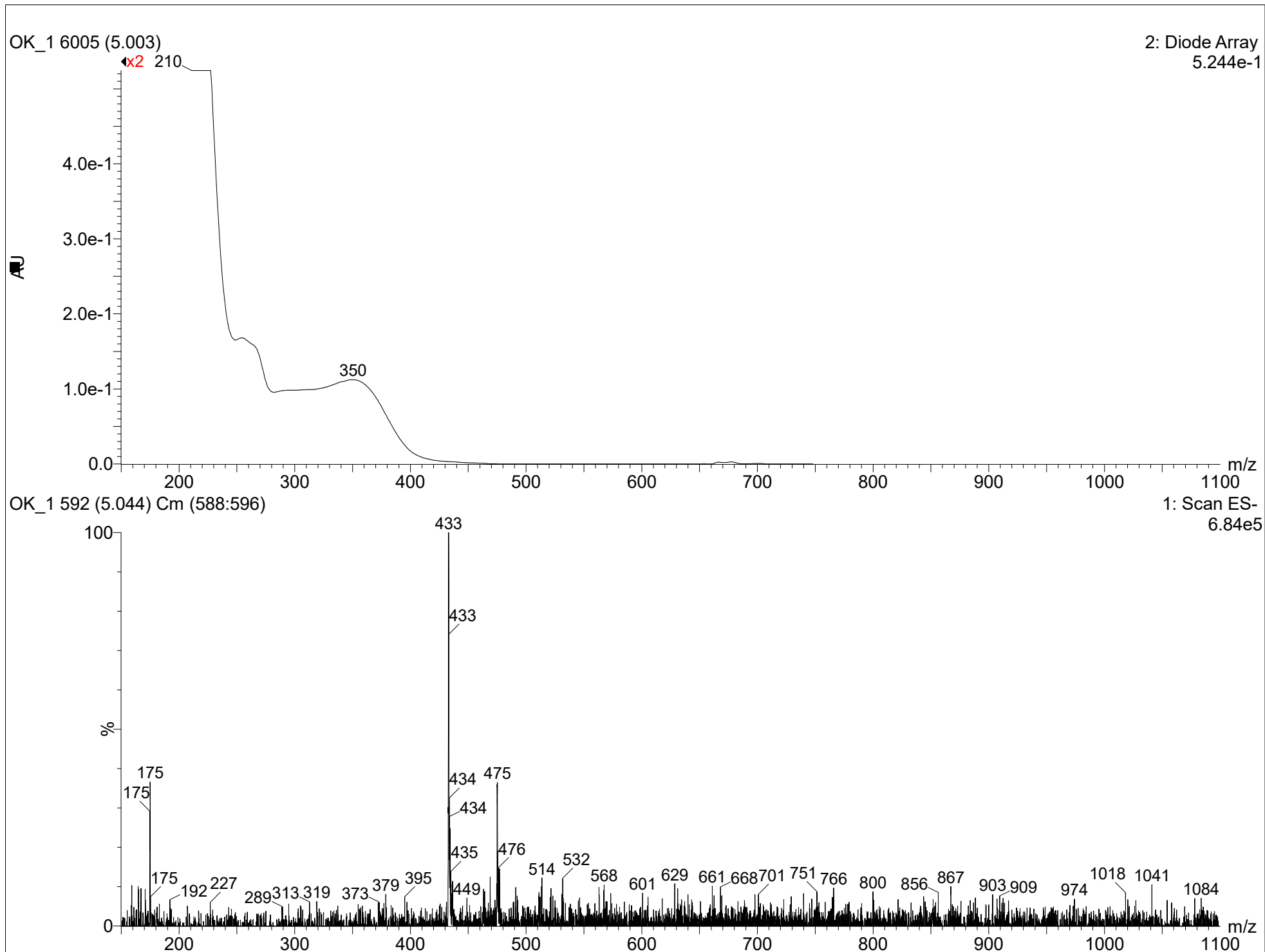

Supplement: Supplementary file 1 [file molecules-27-02762-s001.zip › Compounds 16,18.pdf]

OK\_1 7509 (6.257)

2: Diode Array  
6.483e-1

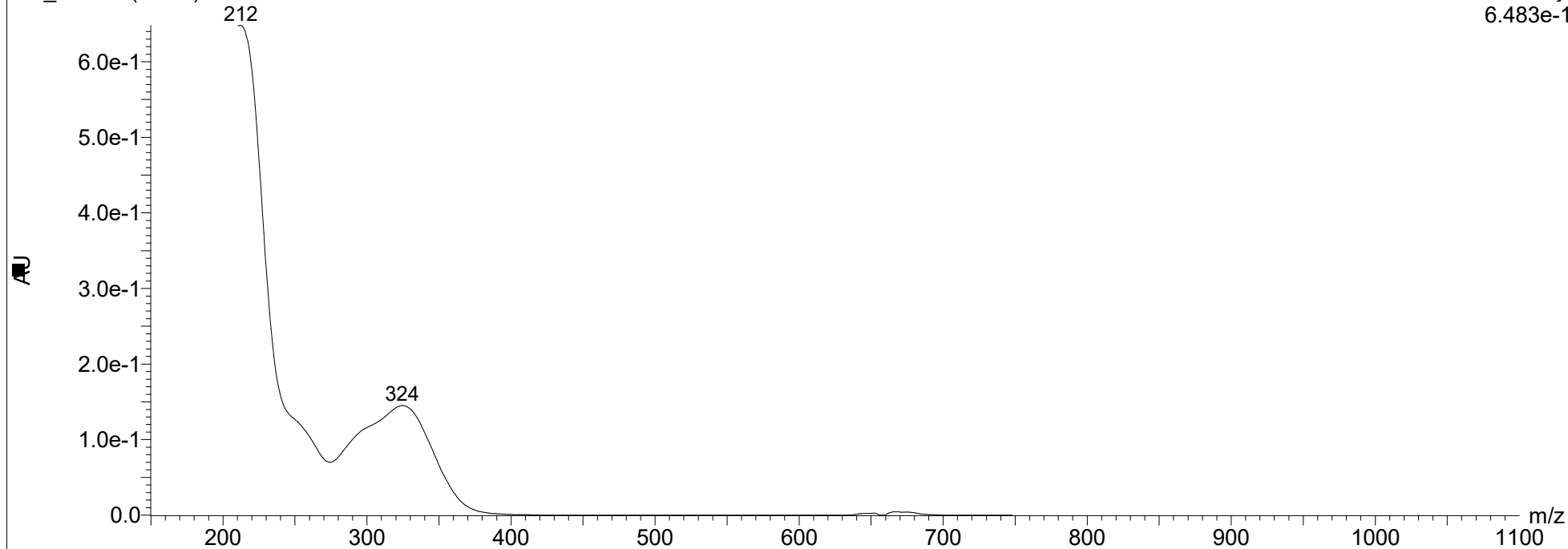

OK\_1 739 (6.296) Cm (734:743)

1: Scan ES-  
1.38e6

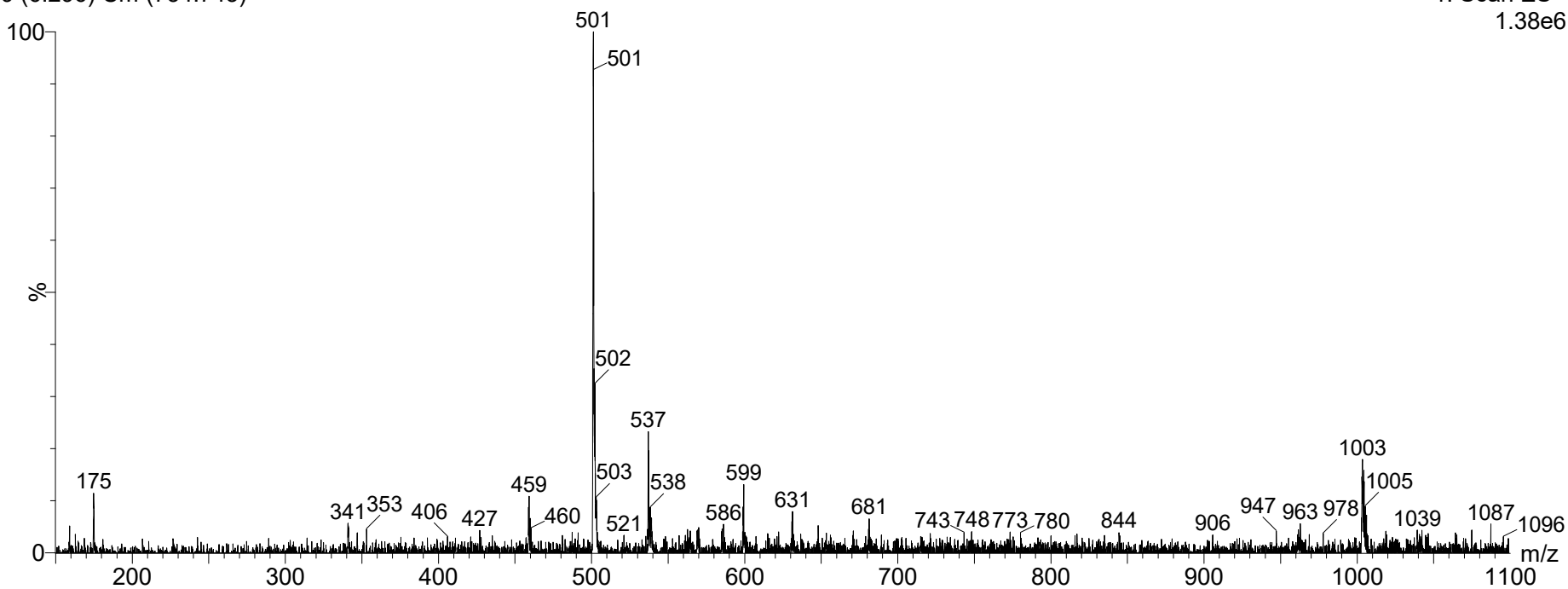

Supplement: Supplementary file 1 [file molecules-27-02762-s001.zip › Compounds 21,24.pdf]

OK\_1 3061 (2.550)

2: Diode Array  
3.57e-1

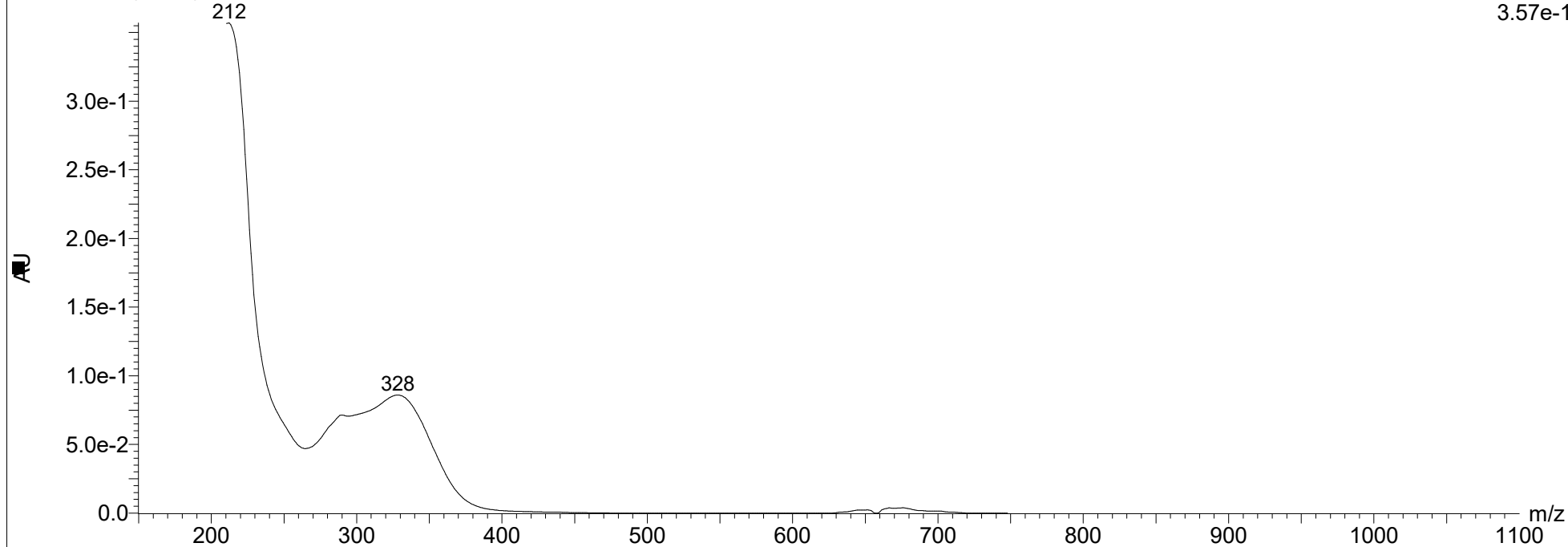

OK\_1 306 (2.607) Cm (301:311)

1: Scan ES-  
5.26e5

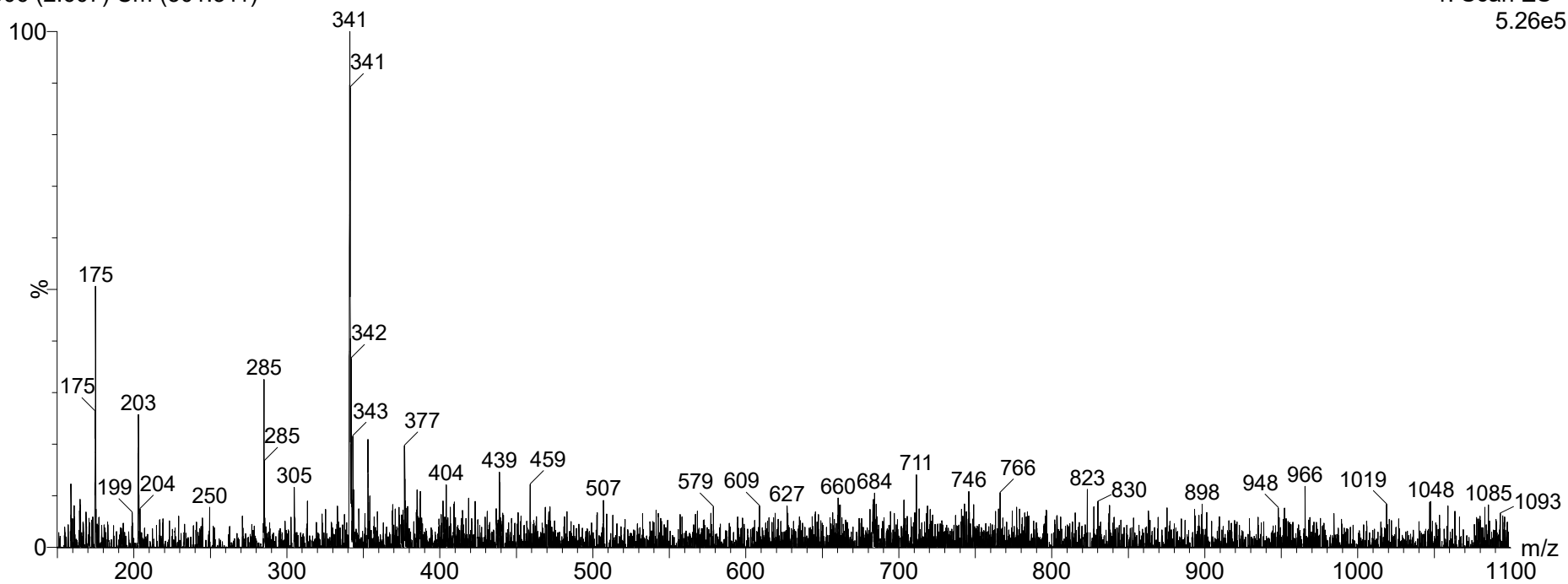

Supplement: Supplementary file 1 [file molecules-27-02762-s001.zip › Compounds 3,4.pdf]

OK\_1 455 (3.877) Cm (452:459)

1: Scan ES-  
7.58e5

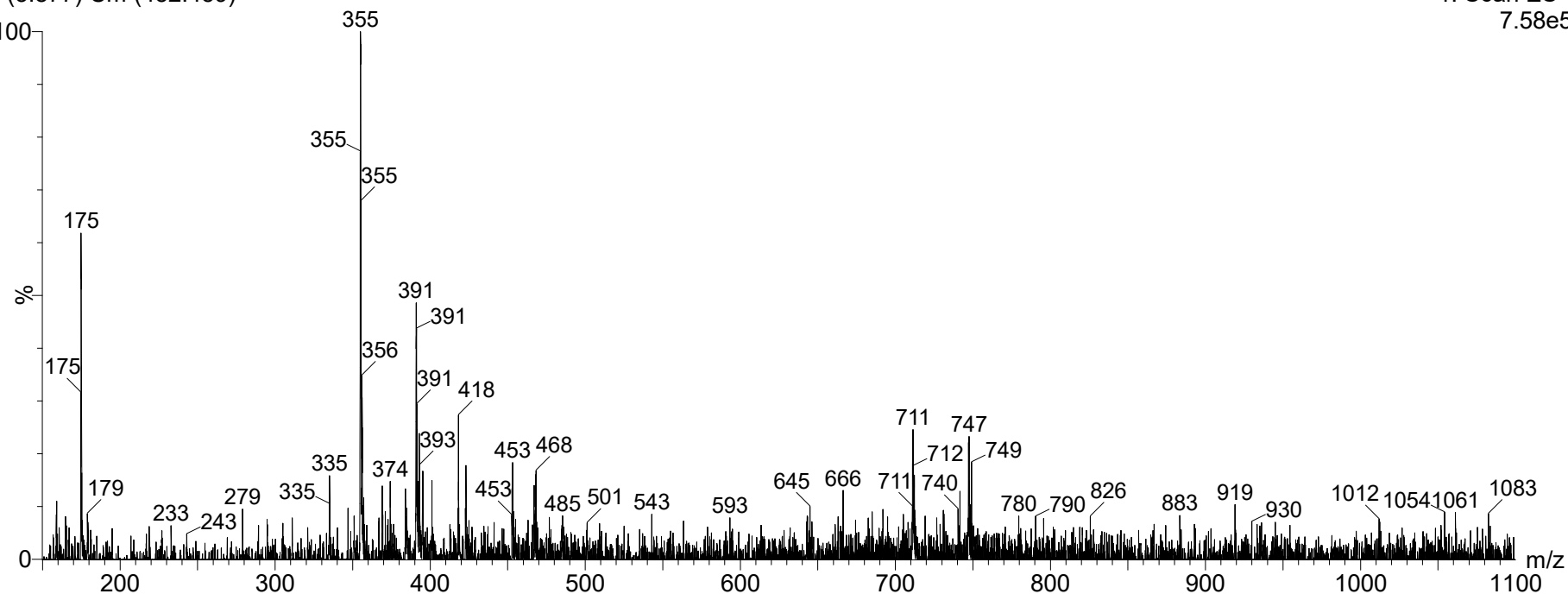

OK\_1 4605 (3.837)

2: Diode Array  
4.601e-1

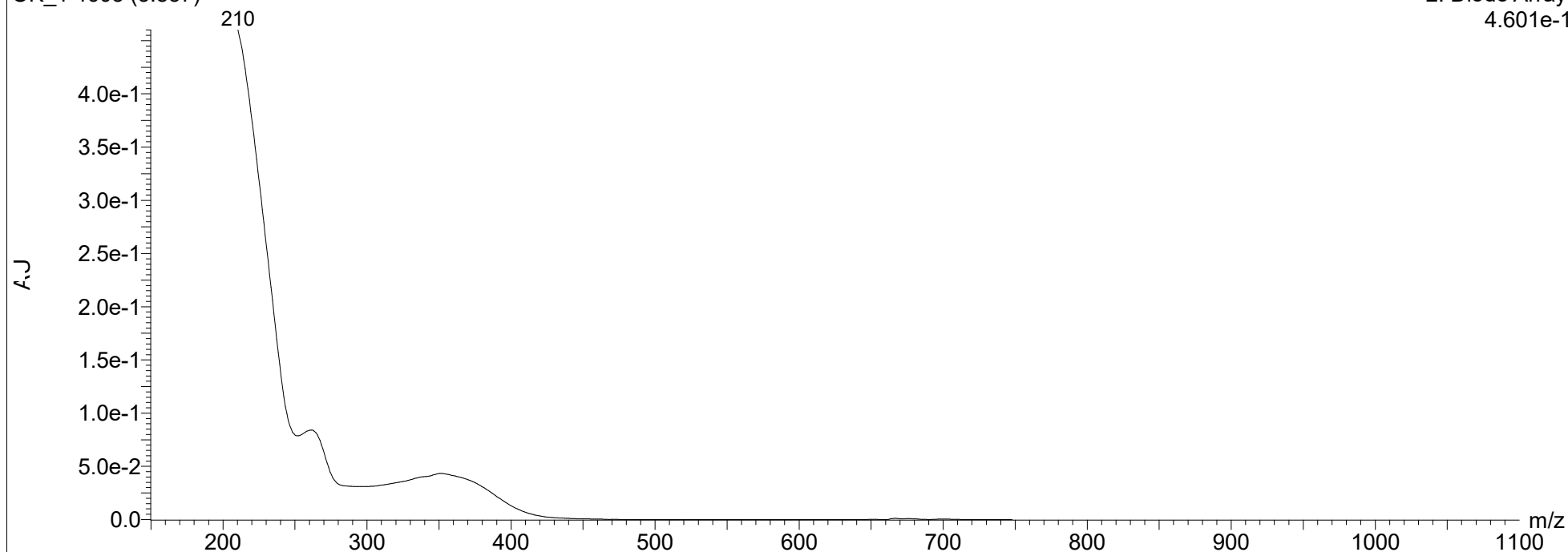

Supplement: Supplementary file 1 [file molecules-27-02762-s001.zip › Compounds 9,11.pdf]

OK\_1 3468 (2.889)

2: Diode Array  
5.698e-1

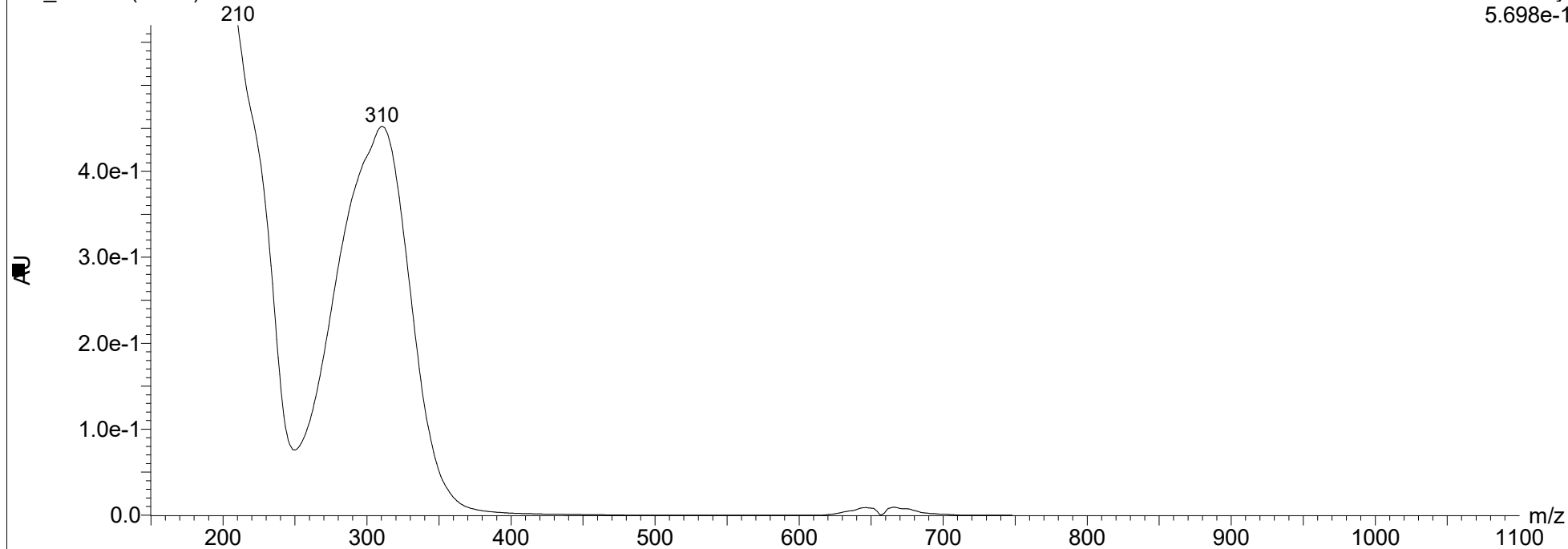

OK\_1 344 (2.931) Cm (342:349)

1: Scan ES-  
1.74e6

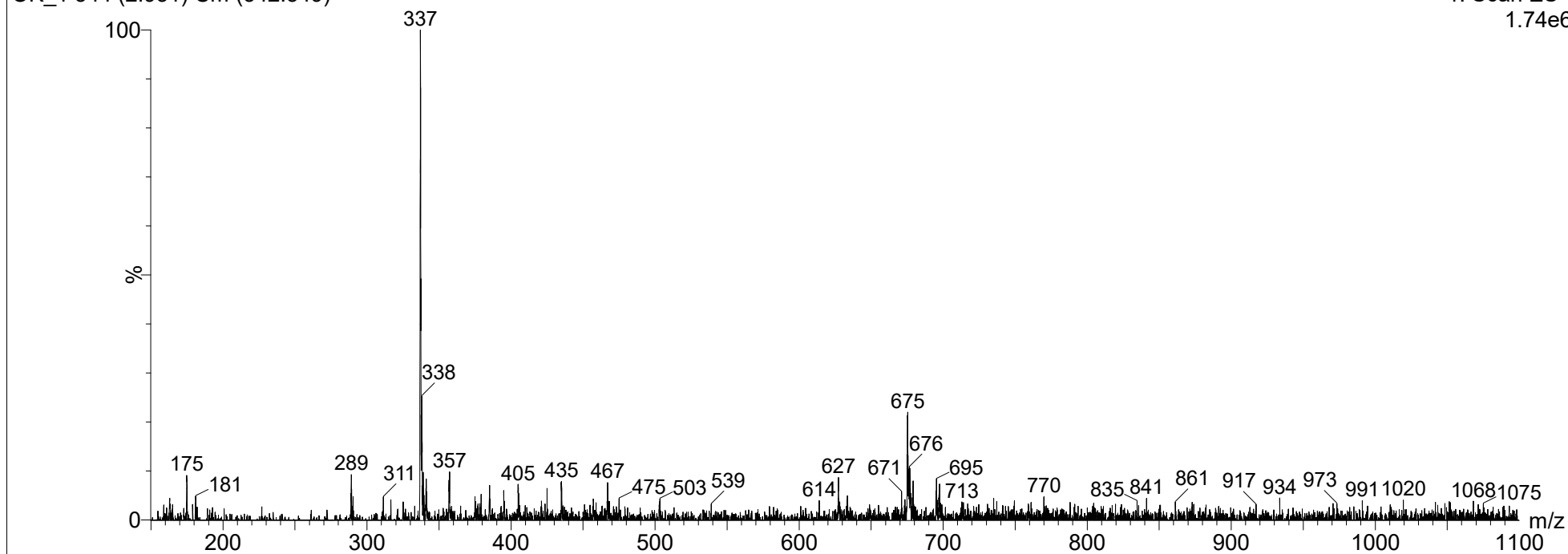

Supplement: Supplementary file 1 [file molecules-27-02762-s001.zip › Compunds 5,10.pdf]
